# MIWI N-terminal RG motif promotes efficient pachytene piRNA production and spermatogenesis independent of LINE1 transposon silencing

**DOI:** 10.1371/journal.pgen.1011031

**Published:** 2023-11-13

**Authors:** Chao Wei, Jiongjie Jing, Xiaoyuan Yan, Jeffrey M. Mann, Ruirong Geng, Huirong Xie, Elena Y. Demireva, Rex A. Hess, Deqiang Ding, Chen Chen

**Affiliations:** 1 Department of Animal Science, Michigan State University, East Lansing, Michigan, United States of America; 2 Shanghai Key Laboratory of Maternal and Fetal Medicine, Clinical and Translational Research Center of Shanghai First Maternity and Infant Hospital, Frontier Science Center for Stem Cell Research, School of Life Sciences and Technology, Tongji University, Shanghai, China; 3 Transgenic and Genome Editing Facility, Institute for Quantitative Health Science & Engineering, Michigan State University, East Lansing, Michigan, United States of America; 4 Department of Comparative Biosciences, University of Illinois, Urbana, Illinois, United States of America; 5 Reproductive and Developmental Sciences Program, Michigan State University, East Lansing, Michigan, United States of America; 6 Department of Obstetrics, Gynecology and Reproductive Biology, Michigan State University, Grand Rapids, Michigan, United States of America; Cornell University, UNITED STATES

## Abstract

PIWI proteins and their associated piRNAs act to silence transposons and promote gametogenesis. Murine PIWI proteins MIWI, MILI, and MIWI2 have multiple arginine and glycine (RG)-rich motifs at their N-terminal domains. Despite being known as docking sites for the TDRD family proteins, the *in vivo* regulatory roles for these RG motifs in directing PIWI in piRNA biogenesis and spermatogenesis remain elusive. To investigate the functional significance of RG motifs in mammalian PIWI proteins *in vivo*, we genetically engineered an arginine to lysine (RK) point mutation of a conserved N-terminal RG motif in MIWI in mice. We show that this tiny MIWI RG motif is indispensable for piRNA biogenesis and male fertility. The RK mutation in the RG motif disrupts MIWI-TDRKH interaction and impairs enrichment of MIWI to the intermitochondrial cement (IMC) for efficient piRNA production. Despite significant overall piRNA level reduction, piRNA trimming and maturation are not affected by the RK mutation. Consequently, *Miwi*^*RK*^ mutant mice show chromatoid body malformation, spermatogenic arrest, and male sterility. Surprisingly, LINE1 transposons are effectively silenced in *Miwi*^*RK*^ mutant mice, indicating a LINE1-independent cause of germ cell arrest distinctive from *Miwi* knockout mice. These findings reveal a crucial function of the RG motif in directing PIWI proteins to engage in efficient piRNA production critical for germ cell progression and highlight the functional importance of the PIWI N-terminal motifs in regulating male fertility.

## Author summary

The presence of RG-rich motifs in the N-terminal region of the PIWI family proteins is a conserved feature. However, how these RG-motifs direct PIWI proteins to participate in piRNA biogenesis and germ cell function *in vivo* remains unknown. Here we genetically mutate a conserved RG motif in MIWI/PIWIL1 in mice to elucidate its physiological function in piRNA regulation and spermatogenesis. For the first time, we show that an RG motif mediating crucial protein-protein interactions is essential for piRNA biogenesis and male fertility in mice. Unexpectedly, this genetic model separates the two defects of the MIWI mutant: spermiogenesis arrest and LINE1 transposon activation. These findings highlight the functional importance of this tiny protein motif in fertility regulation.

## Introduction

PIWI proteins are germline-specific Argonaute proteins that play pivotal roles in genome defense and gametogenesis [1–5]. They function by associating with PIWI-interacting RNAs (piRNAs) to silence transposons and regulate germline genes through a small RNA-guided regulatory pathway [6–10]. Impairment of PIWI proteins and piRNAs results in transposon dysregulation, germ cell developmental arrest, and infertility [11–16].

In mice, three PIWI proteins, MIWI, MILI, and MIWI2 (also known as PIWIL1, PIWIL2, and PIWIL4, respectively), are expressed during male germ cell development and all three are essential for spermatogenesis [3]. MILI and MIWI2, having a primary function in transposon silencing in embryonic germ cells, are associated with fetal piRNAs [14,17–19]. In postnatal germ cells, MIWI and MILI associate with piRNAs in pachytene spermatocytes and round spermatids to regulate meiosis, spermiogenesis, as well as suppressing transposons [20–24]. All three PIWI proteins share conserved protein domains (MID, PAZ, and PIWI) with well-defined functions. MID and PAZ domains function to bind the piRNA 5’- and 3’-ends, respectively [25,26]. The PIWI domain forms an RNaseH-like fold and exhibits RNA slicing activity [18,27]. A variable N-terminal domain, common to all PIWI proteins, is least functionally characterized and contains multiple RG-rich motifs that are subject to arginine methylation [28–32]. These RG motifs are known to serve as binding sites for a group of Tudor domain-containing proteins (TDRDs) [31,32]. However, how these RG motifs direct PIWI proteins to engage in the piRNA pathway to regulate spermatogenesis remain elusive.

Pachytene piRNAs are generated by the piRNA biogenesis machinery from precursor transcripts derived from discrete genomic loci at the onset of meiosis [33]. While pachytene piRNAs contain a tiny fraction of transposon-related piRNAs, the overwhelming majority are non-transposon piRNAs derived from intergenic piRNA clusters. The biological function of these pachytene piRNAs still lacks consensus [21–23,34–38]. Concomitant with pachytene piRNA biogenesis, MILI and MIWI are sequentially expressed and eventually loaded with almost the same sets of pachytene piRNAs [20,39]. Unlike MILI, MIWI is not expressed in embryonic germ cells but is exclusively bound to meiotic pachytene piRNAs, starting from mid-pachytene spermatocytes to round spermatids [11,33]. MIWI plays critical roles in transposon silencing and germ cell differentiation as *Miwi* knockout mice show drastically upregulated LINE1 transposons in spermatocytes and round spermatids and display germ cell arrest at the round spermatid step of spermiogenesis [11,27]. MIWI slicer activity is required for LINE1 silencing as well as spermatid differentiation [11,27]. Despite MIWI’s important roles, it remains unclear whether transposon dysregulation causes germ cell arrest due to MIWI deficiency.

Here we use a conserved RG motif of MIWI as a prototype to study the *in vivo* function of RG motifs in mammalian PIWI proteins. We discovered that the MIWI N-terminal RG motif is crucial for MIWI function and spermatogenesis. Mutations in the N-terminal RG motif disrupt specific MIWI-TDRD interactions and impair pachytene piRNA biogenesis, resulting in male sterility. Importantly, the RG motif mutation separates the two defects of MIWI ablation: spermiogenesis arrest and LINE1 transposon activation.

## Results

### MIWI N-terminal RG motif selectively interacts with TDRKH *in vitro*

**Fig 1:**
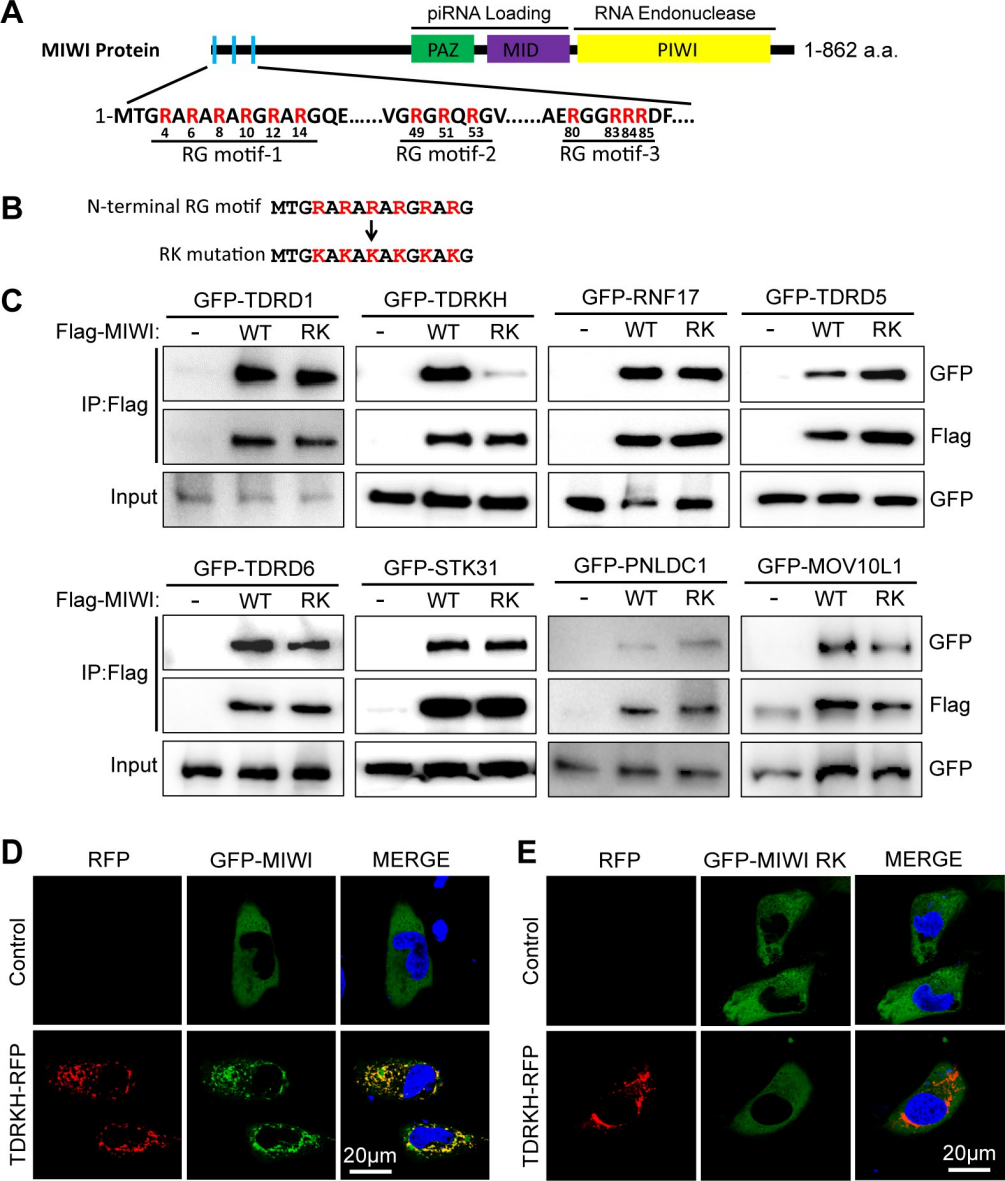
MIWI N-terminal RG motif selectively interacts with TDRKH. **(A)** Schematic illustration of the domain architecture of mouse MIWI. **(B)** Protein sequences of the N-terminal RG motif (RG motif-1 in (A)) and its RK mutation are shown. **(C)** The RK mutation (RK) in MIWI N-terminal RG motif selectively diminishes the interaction of MIWI with TDRKH. HEK293T cells were transfected with indicated plasmids. Immunoprecipitation was performed using anti-FLAG resin. GFP-tagged and FLAG-tagged proteins were detected by Western blotting with anti-GFP and anti-FLAG antibodies. **(D)** TDRKH recruits MIWI to mitochondria. HeLa cells were transfected with GFP-tagged MIWI alone or together with RFP-tagged TDRKH plasmids. After 48 h, the cells were fixed and DNA was stained with DAPI. Scale bar, 20 μm. **(E)** The RK mutation diminishes the recruitment MIWI to mitochondria by TDRKH. HeLa cells were transfected with GFP-tagged MIWI RK alone, or together with RFP-tagged TDRKH plasmids. After 48 h, cells were fixed and DNA was stained with DAPI. Scale bar, 20 μm. Results shown in (C)-(E) are representative of 3 biological replicates.

The N-terminal domain of MIWI contains three RG motifs as TDRD protein binding sites (Fig 1A). We previously reported that TDRKH (TDRD2) specifically interacts with MIWI through the RG motif-1 (termed MIWI N-terminal RG motif thereafter) but not through RG motif-2 or RG motif-3, and this interaction is arginine methylation-independent [28,40]. Since MIWI interacts with TDRD proteins and other piRNA biogenesis factors [30], we tested whether MIWI N-terminal RG motif also mediates interaction with other proteins. To abolish arginine-mediated protein interactions (methylation-dependent and methylation-independent), we constructed a *Miwi* arginine to lysine (*Miwi*^*RK*^) mutation in which all six arginines (R) in the N-terminal RG motif were mutated to lysines (K) (Fig 1B). This mutation is not expected to change the charge state of MIWI^RK^ mutant protein (MIWI^RK^) and therefore minimally impacts the biophysical property of MIWI. Co-expression of FLAG-tagged MIWI or MIWI^RK^ with different GFP-tagged piRNA pathway proteins in HEK293T cells were used to assess MIWI protein interactions by immunoprecipitation and Western blotting. While MIWI interacted with all previously reported MIWI-interacting proteins including TDRD1, TDRKH, RNF17 (TDRD4), TDRD5, TDRD6, STK31 (TDRD8), PNLDC1 and MOV10L1, the RK mutation significantly disrupted the interaction of MIWI^RK^ with TDRKH (Fig 1C). The interaction of MIWI^RK^ with other proteins was minimally affected (Fig 1C). TDRKH is a mitochondrial membrane anchored protein capable of recruiting cytoplasmic MIWI to mitochondria [41,42]. Since the *Miwi*^*RK*^ mutation strongly disrupted MIWI-TDRKH interaction, we reasoned that MIWI^RK^ would fail to be recruited to mitochondria by TDRKH. We expressed GFP-MIWI or GFP-MIWI^RK^ in HeLa cells, both showing diffused distribution in cytoplasm when singly transfected. When co-expressed with TDRKH-RFP, GFP-MIWI concentrated on mitochondria to significantly overlap with mitochondrial TDRKH-RFP (Fig 1D). However, GFP-MIWI^RK^ still displayed diffused cytoplasmic distribution in the presence of TDRKH-RFP, indicating failure of recruitment by TDRKH to mitochondria (Fig 1E). Together, these data demonstrate that MIWI N-terminal RG motif is required for specific MIWI-TDRKH interaction and regulates MIWI localization to mitochondria to engage in piRNA biogenesis.

### RK mutation in MIWI N-terminal RG motif disrupts spermatogenesis in mice

**Fig 2:**
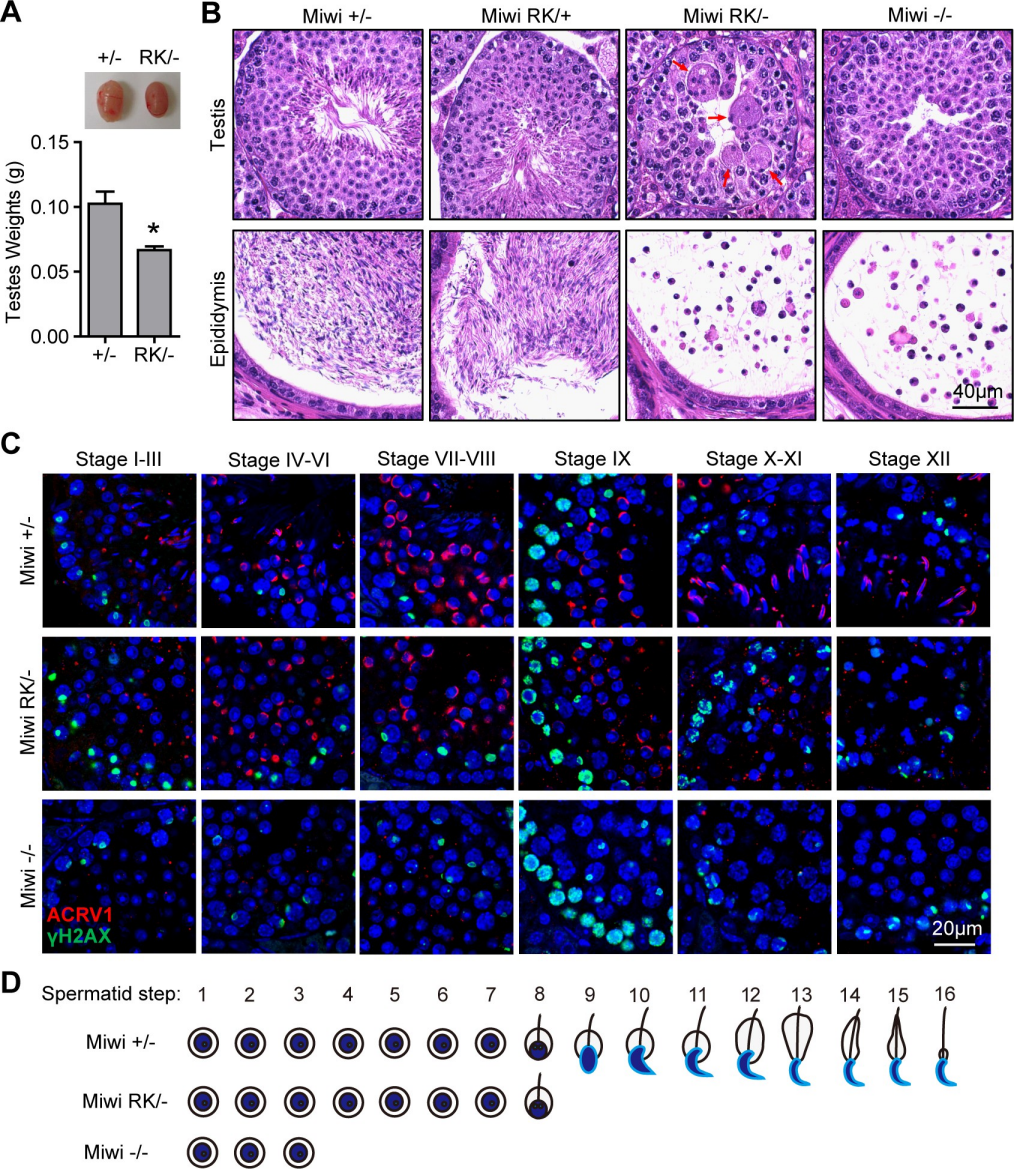
MIWI N-terminal RG motif is essential for spermatogenesis. **(A)** Testicular atrophy in *Miwi*^*RK/-*^ mice. Testis sizes and weights of adult *Miwi*^*+/-*^ and *Miwi*^*RK/-*^ mice are shown. n = 6. Error bars represent s.e.m. The P-value was calculated using unpaired t-test. *, P < 0.01. **(B)** The RK mutation in MIWI N-terminal RG motif causes spermatogenic arrest in mice. H&E staining of testes and epididymides from *Miwi*^*+/-*^, *Miwi*^*RK/+*^, *Miwi*^*RK/-*^ and *Miwi*^*-/-*^ mice are shown. The multinucleated giant cells in *Miwi*^*RK/-*^ testes are indicated by red arrows. Scale bar, 40 μm. **(C)** Testes were co-immunostained using ACRV1 and γH2AX antibodies. DNA was stained by DAPI. Scale bar, 20 μm. **(D)** A cartoon illustrating the arrested spermiogenic steps in *Miwi*^*RK/-*^ and *Miwi*^*-/-*^ mice. Results shown in (B) and (C) are representative of 3 biological replicates.

The *in vivo* function of RG motifs of mammalian PIWI proteins is unknown. We sought to use the MIWI N-terminal RG motif as a prototype to study the functional significance of RG motifs of PIWI proteins during piRNA biogenesis and spermatogenesis in mice (S1 Fig). Using CRISPR-Cas9 genome editing, we generated a *Miwi*^*RK*^ mutant allele in mice in which all six arginines in MIWI N-terminal RG motif were mutated to lysines (S1B Fig). We confirmed these mutations in *Miwi*^*RK*^ mutant mice by Sanger sequencing (S1C Fig). By breeding the *Miwi*^*RK*^ allele into *Miwi* null background (*Miwi*^*RK/-*^), we next examined whether the *Miwi*^*RK*^ mutation affects spermatogenesis. *Miwi*^*RK/-*^ mice are viable and grow normally but exhibited reduced testis mass compared to *Miwi*^*RK/+*^ and *Miwi*^*+/-*^ control littermates (Fig 2A). Notably, *Miwi*^*RK/+*^ male mice are fertile, indicating that the *Miwi*^*RK*^ mutation does not have a dominant-negative effect on germ cell development (Fig 2B). This sharply contrasts to the observed dominant-negative effect of the *Miwi* catalytically inactivating mutation (slicer mutation) on spermatogenesis, suggesting that the RK mutation did not affect MIWI catalytic activity [27]. Histological examination of *Miwi*^*RK/-*^ testes revealed that germ cells were arrested at the round spermatid stage and no elongating spermatids were formed (Fig 2B). As a result, numerous round spermatid-like cells were accumulated in epididymides of *Miwi*^*RK/-*^ mice (Fig 2B). Together, these data demonstrate that genetic mutation in MIWI N-terminal RG motif blocks spermatogenesis and causes male sterility.

### *Miwi*^*RK/-*^ and *Miwi*^*-/-*^ mice display distinct spermiogenic arrests

Interestingly, we observed multinucleated giant cells in about half of *Miwi*^*RK/-*^ seminiferous tubules which were rarely seen in *Miwi*^*-/-*^ tubules (Fig 2B). This difference suggests that germ cells were arrested at distinctive round spermatid stages in *Miwi*^*RK/-*^ and *Miwi*^*-/-*^ testes. To verify this, we performed immunostaining of ACRV1, an acrosome marker, in *Miwi*^*+/-*^, *Miwi*^*RK/-*^ and *Miwi*^*-/-*^ testes. Acrosomes formed normally until step 8 spermatids which failed to elongate further in *Miwi*^*RK/-*^ testes. By contrast, acrosome formation was almost completely blocked in *Miwi*^*-/-*^ spermatids, indicating that round spermatids were arrested at steps 1–3 in *Miwi*^*-/-*^ testes (Fig 2C and 2D). This suggests that the RK mutation in MIWI blocks the function of MIWI to fully promote spermiogenesis.

We next explored the nature of the presence of multinucleated giant cells in *Miwi*^*RK/-*^ testes. We speculated that multinucleated giant cells in *Miwi*^*RK/-*^ testes were being removed by Sertoli cells by phagocytosis, because the giant cells were not observed in the cauda epididymis. To test this, we performed immunostaining of α-tubulin that is highly expressed in Sertoli cell cytoplasm and found that multinucleated giant cells were surrounded by Sertoli cell cytoplasm in *Miwi*^*RK/-*^ testes (S2 Fig). This suggests that the formation of multinucleated giant cells is caused by phagocytic removal of spermatogenic arrested cells by Sertoli cells in *Miwi*^*RK/-*^ testes.

### MIWI^RK^ fails to interact with TDRKH to localize to IMC despite normal initial expression

**Fig 3:**
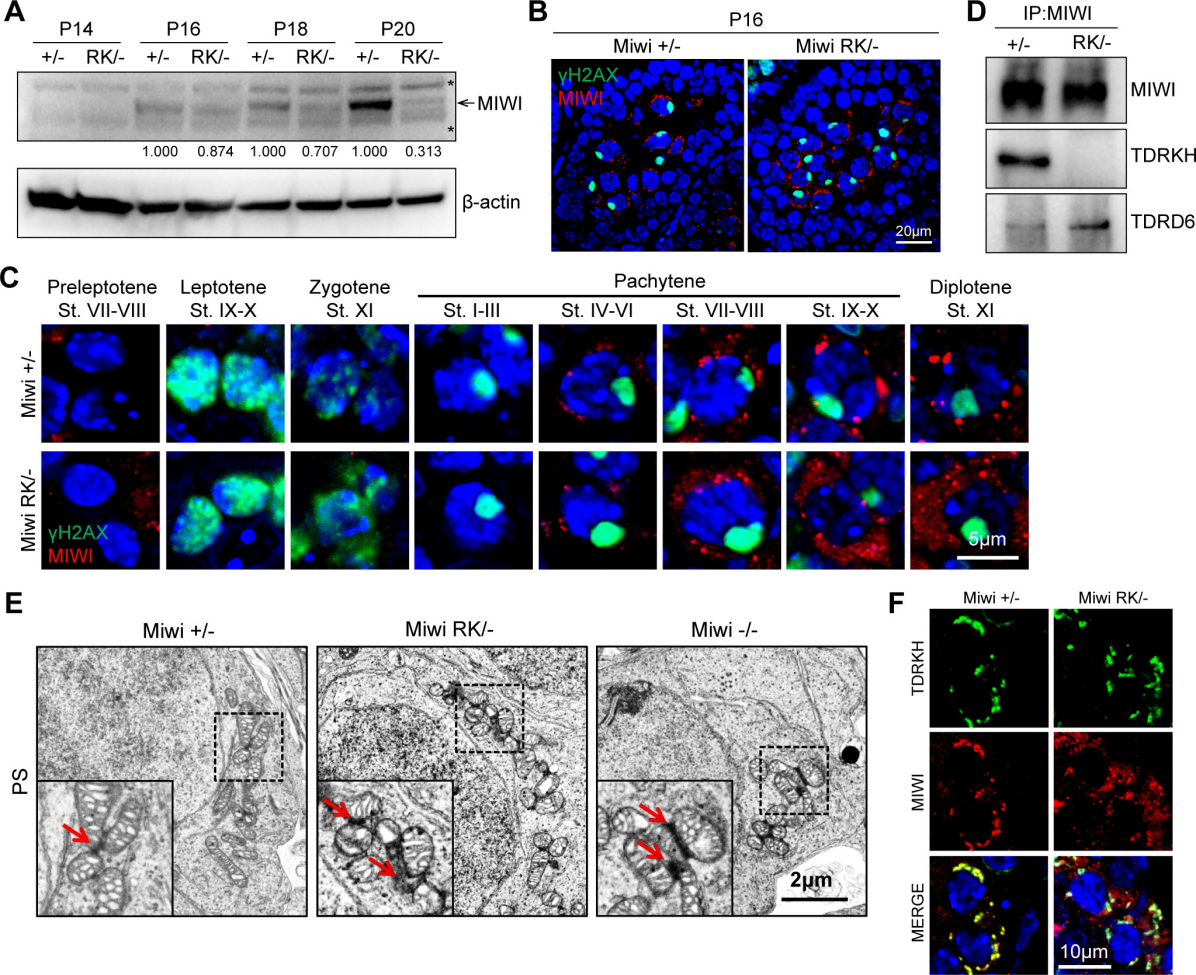
The N-terminal RG motif is required for recruitment of MIWI to the IMC in spermatocytes. **(A)** Western blotting of MIWI at different postnatal (P) ages *Miwi*^*+/-*^ and *Miwi*^*RK/*-^ testes. β-actin is a loading control. Asterisks indicate non-specific bands. Quantification of intensity of MIWI is shown under the blot (the one in *Miwi*^*+/-*^ testis of each group is set as 1.000 after normalization with β-actin). **(B)** Testes from P16 mice were co-immunostained using MIWI and γH2AX antibodies. DNA was stained by DAPI. Scale bar, 20 μm. **(C)** The RK mutation causes mislocalization of MIWI in late spermatocytes. Testes from indicated mice were immunostained using MIWI and γH2AX antibodies. DNA was stained by DAPI. Different cell types were distinguished according to γH2AX staining and DAPI staining. Scale bar, 5 μm. **(D)** The RK mutation selectively disrupts the interaction of MIWI with TDRKH. Immunoprecipitation was performed using anti-MIWI antibody. MIWI, TDRKH and TDRD6 were detected by Western blotting. **(E)** The RK mutation in MIWI does not affect the formation of the IMC in spermatocytes. Transmission electron microscopy was performed on pachytene spermatocytes (PS) from indicated testes. The IMC region is zoomed and indicated by red arrows. Scale bar, 2 μm. **(F)** The RK mutation diminishes the recruitment of MIWI to mitochondria in pachytene spermatocytes. Co-immunostaining of MIWI and TDRKH in stage VII–VIII seminiferous tubule from *Miwi*^*+/-*^ and *Miwi*^*RK/*-^ testes was performed. DNA was stained by DAPI. Scale bar, 10 μm. Results shown in (A)-(F) are representative of 3 biological replicates.

We next examined the effect of the RK mutation on MIWI mutant protein (MIWI^RK^) expression and localization during spermatogenesis. As expected, MIWI^RK^ in *Miwi*^*RK/-*^ testes started to express in early pachytene spermatocytes, resembling the timing of wild-type MIWI (Fig 3A). At P16, MIWI^RK^ expression level and localization pattern in *Miwi*^*RK/-*^ testes was similar to that of wild-type MIWI in *Miwi*^*+/-*^ testes, indicating that the RK mutation does not affect the initial expression and stability of MIWI (Fig 3A and 3B). However, MIWI^RK^ displayed lower expression levels than wild-type MIWI in P18 and P20 testes as meiosis progresses (Fig 3A). In adult mice, MIWI^RK^ in *Miwi*^*RK/RK*^ and *Miwi*^*RK/-*^ testes was expressed at a lower level than total MIWI in *Miwi*^*RK/+*^ and *Miwi*^*+/-*^ testes, respectively (S3A Fig). We observed a diffused distribution of MIWI^RK^ in mid-late pachytene and diplotene spermatocytes (Figs 3C and S3A). MILI and TDRKH subcellular localization and expression levels, on the other hand, did not change in *Miwi*^*RK/-*^ germ cells (S4 Fig). The observed MIWI^RK^ mislocalization correlates with the timing of pachytene piRNA production and the initial interaction with TDRKH. To further confirm the MIWI-TDRKH interaction is abolished by the RK mutation *in vivo*, we performed co-immunoprecipitation and Western blotting and showed that MIWI^RK^ failed to interact with TDRKH in *Miwi*^*RK/-*^ testes (Fig 3D). As a control, the interaction of MIWI^RK^ with TDRD6 remained intact (Fig 3D), recapitulating our *in vitro* observation (Fig 1C) and suggesting the RK mutation selectively disrupts MIWI-TDRKH interaction *in vivo*. Transmission electron microscopy showed the IMC formation in *Miwi*^*RK/-*^ spermatocytes is largely normal, undistinguishable from that in *Miwi*^*+/-*^ or *Miwi*^*-/-*^ (Fig 3E). We further co-stained MIWI with TDRKH or mitochondrial marker TOMM20 in stage VII-VIII pachytene spermatocytes. While MIWI and TDRKH/TOMM20 completely colocalized at the IMC in *Miwi*^*+/-*^ control, MIWI^RK^ only slightly overlapped with TDRKH/TOMM20 at the IMC in *Miwi*^*RK/-*^ spermatocytes, with most of its distribution in the cytoplasm (Figs 3F and S3B). This suggests that the efficiency of MIWI^RK^ being recruited by TDRKH to the IMC is severely compromised, which can be explained by the diminished MIWI^RK^-TDRKH interaction observed in *Miwi*^*RK/-*^ testes (Fig 3D). Collectively, the RK mutation did not affect MIWI^RK^ initial expression. Subsequently altered MIWI^RK^ expression and localization are likely due to the loss of MIWI RG motif-mediated protein-protein interactions.

### The N-terminal RG motif of MIWI regulates the efficiency of pachytene piRNA biogenesis

**Fig 4:**
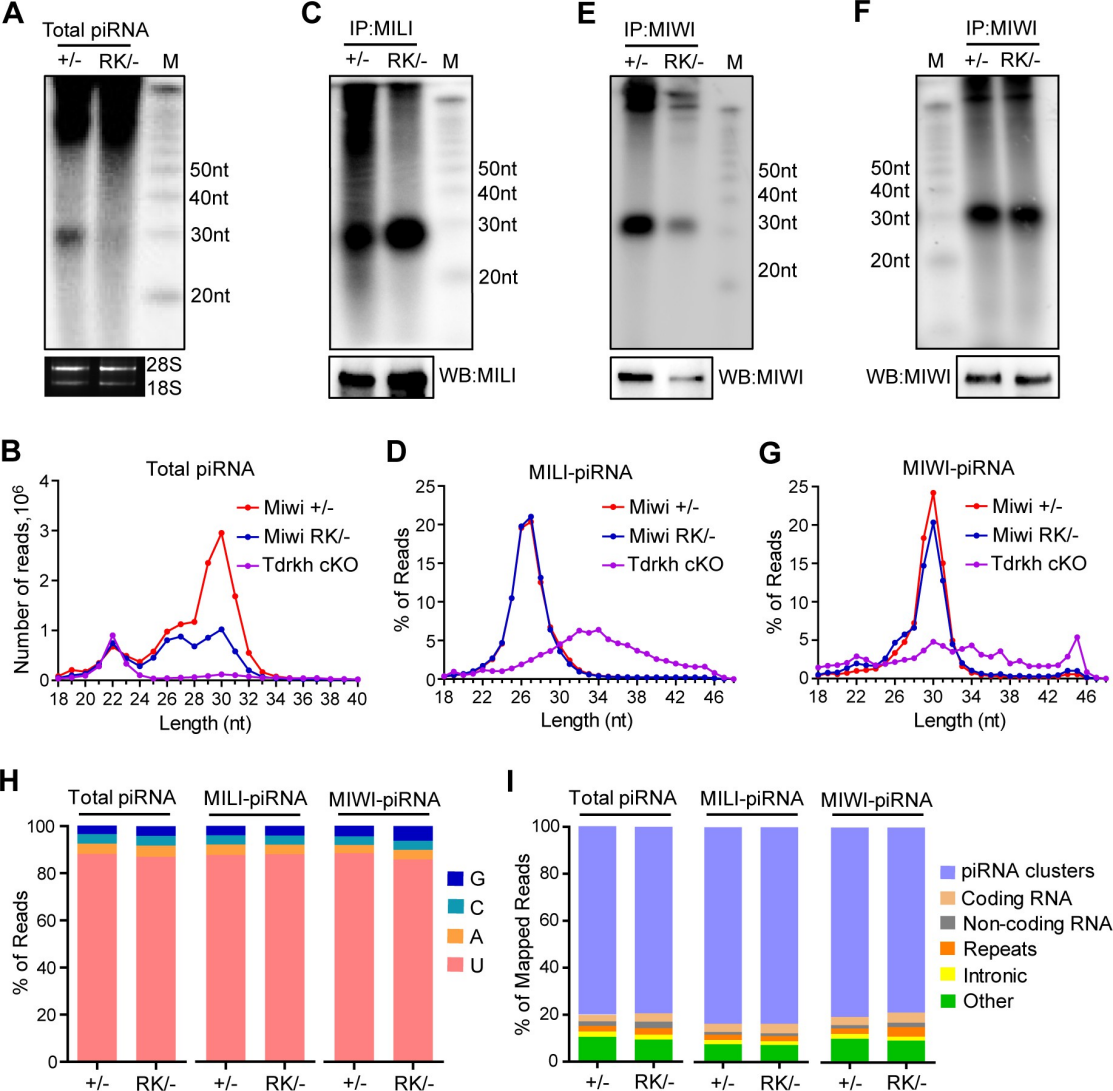
MIWI N-terminal RG motif is required for efficient pachytene piRNA biogenesis. **(A)** The RK mutation in MIWI N-terminal RG motif causes reduction of total piRNA in adult testes. Total RNA from testes of indicated genotypes were end-labeled with [32P]-ATP and detected by TBE urea gel and autoradiography. 18S and 28S ribosomal RNAs served as loading controls. **(B)** The length distribution of small RNAs from adult testes. Data were normalized by miRNA reads (21–23nt). **(C)** The RK mutation in MIWI does not affect MILI-piRNA biogenesis. Small RNAs were isolated from immunoprecipitated MILI-RNPs, end-labeled with [32P]-ATP and detected by TBE urea gel and autoradiography. Western blotting was performed using anti-MILI antibody after immunoprecipitation. **(D)** The length distribution of MILI-piRNAs from adult testes of indicated genotypes. **(E)** The RK mutation in MIWI causes reduction in MIWI-piRNAs. Small RNAs were isolated from immunoprecipitated MIWI-RNPs, end-labeled with [32P]-ATP and detected by TBE urea gel and autoradiography. Western blotting was performed using anti-MIWI antibody after immunoprecipitation. **(F)** MIWI^RK^ are properly loaded with piRNAs. Small RNAs were isolated from immunoprecipitated MIWI-RNPs, end-labeled with [32P]-ATP and detected by TBE urea gel and autoradiography. MIWI protein levels after immunoprecipitation served as loading controls. **(G)** The length distribution of MIWI-piRNAs from adult testes of indicated genotypes. **(H)** Nucleotide distributions at the first position in total piRNA, MILI-piRNAs and MIWI-piRNAs from adult testes of indicated genotypes. **(I)** Genomic annotation of total piRNA, MILI-piRNAs and MIWI-piRNAs from adult testes of indicated genotypes are shown. Sequence reads from indicated libraries were aligned to mouse genomic sequence sets in the following order: piRNA clusters, coding RNA, non-coding RNA, repeats, intronic sequences and other. Results shown in (A), (C), (E) and (F) are representative of 3 biological replicates. Small RNA-seq results shown in (B), (D), (G), (H) and (I) are representative of 2 biological replicates.

We then asked how the RK mutation affects MIWI in participating in piRNA production. We examined the abundance and size of piRNA populations in adult *Miwi*^*+/-*^ and *Miwi*^*RK/-*^ testes. Radiolabeling of total RNA revealed that total piRNA production was much reduced in *Miwi*^*RK/-*^ testes (Fig 4A). We further sequenced these small RNAs from total RNA to show two piRNA populations corresponding to 25-28nt MILI-piRNAs and 29-32nt MIWI-piRNAs in *Miwi*^*+/-*^ and *Miwi*^*RK/-*^ testes. After normalizing to miRNA counts (21-23nt), the abundance of MILI-piRNAs was unaffected. However, the level of MIWI-piRNAs was significantly reduced in *Miwi*^*RK/-*^ testes (Fig 4B). Despite this, the overall piRNA reduction was moderate compared with the severe piRNA loss in Stra8-Cre *Tdrkh* conditional knockout (*Tdrkh*^*cKO*^) mice (Fig 4B). To confirm this trend, we analyzed MILI-piRNAs and MIWI-piRNAs by immunoprecipitation of MILI and MIWI followed by RNA labeling. MILI-piRNAs had the same abundance and lengths in *Miwi*^*RK/-*^ testes compared to the *Miwi*^*+/-*^ control (Fig 4C). Sequencing of MILI-piRNAs further confirmed that MILI-piRNA lengths were normal in *Miwi*^*RK/-*^ testes, a clear difference from the extended untrimmed MILI-piRNAs observed in *Tdrkh*^*cKO*^ testes (Fig 4D). In contrast, MIWI-piRNAs were significantly decreased in *Miwi*^*RK/-*^ testes (Fig 4E). Notably, MIWI level from immunoprecipitation was also reduced in *Miwi*^*RK/-*^ testes (Fig 4E). To verify that the piRNA binding ability of MIWI^RK^ was not altered, MIWI immunoprecipitation was re-performed using the same amount of immunoprecipitated MIWI and MIWI^RK^ for RNA labeling. MIWI^RK^ was fully piRNA-loaded in *Miwi*^*RK/-*^ testes compared to wild-type MIWI, suggesting that the RK mutation does not change the piRNA binding activity of MIWI^RK^ (Fig 4F). Sequencing of MIWI-piRNAs in *Miwi*^*RK/-*^ testes confirmed that MIWI-piRNA lengths were not affected, indicating that the direct MIWI-TDRKH interaction is not required for piRNA trimming (Fig 4G).

Further analysis of *Miwi*^*RK/-*^ piRNAs revealed a strong 5’-end U-bias at the first nucleotides, suggesting that piRNA 5’ formation is normal in *Miwi*^*RK/-*^ mice (Fig 4H). When mapping piRNA reads to the mouse genome, total piRNA, MILI-piRNAs, and MIWI-piRNAs from *Miwi*^*RK/-*^ testes were primarily mapped to piRNA clusters, resembling the wild-type distribution (Fig 4I). The decrease in piRNA amount in *Miwi*^*RK/-*^ testes distributed evenly across top pachytene piRNA producing loci (S5 Fig). This indicates that the RK mutation does not affect the selection of piRNA precursors for processing. Taken together, these data demonstrate for the first time that the MIWI N-terminal RG motif is required for piRNA biogenesis, and the RK mutation results in decreased efficiency of piRNA production without impacting piRNA loading and maturation.

### LINE1 is properly suppressed in *Miwi*^*RK/-*^ germ cells

**Fig 5:**
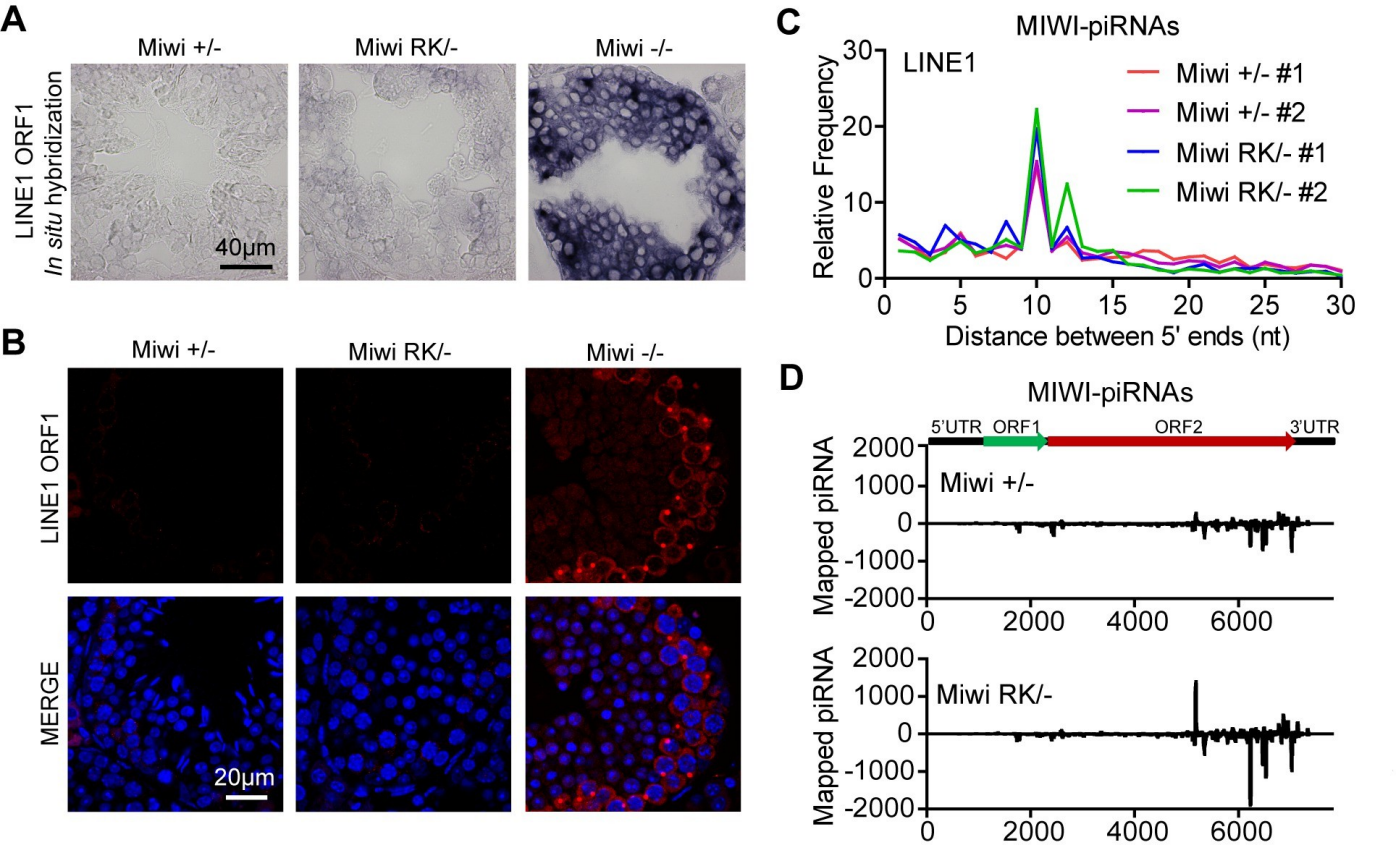
MIWI RK mutation does not cause LINE1 de-repression. **(A)** LINE1 ORF1 mRNA was upregulated in *Miwi*^*-/-*^ testes but was undetectable in *Miwi*^*RK/-*^ testes. *In situ* hybridization of LINE1 ORF1 mRNA was performed in adult testes. Scale bar, 40 μm. **(B)** LINE1 ORF1 protein was upregulated in *Miwi*^*-/-*^ testes but was undetectable in *Miwi*^*RK/-*^ testes. Immunostaining was performed using LINE1 ORF1 antibody on adult testes. DNA was stained with DAPI. Scale bar, 20 μm. **(C)** The MIWI RK mutation does not affect the Ping-Pong signature of LINE1-derived piRNAs. The 5′-5′ overlaps (Ping-Pong signature) between MIWI-piRNAs from opposite strands of LINE1 elements from *Miwi*^*+/-*^ and *Miwi*^*RK/-*^ testes were shown. The percentage of pairs of piRNA reads at each position is reported. **(D)** Graphs showing the distribution of MIWI-piRNAs that were mapped in the sense and antisense orientations to LINE1. Reads were normalized with the total reads from each library. Results shown in (A) and (B) are representative of 3 biological replicates. Small RNA-seq results shown in (D) are representative of 2 biological replicates.

MIWI is required for LINE1 transposon silencing during spermatogenesis [27]. Loss of MIWI causes LINE1 de-repression in pachytene spermatocytes and round spermatids, and MIWI slicer activity is pivotal for LINE1 suppression [27]. We then asked whether the MIWI N-terminal RG motif is required for LINE1 regulation. As previously reported, *in situ* hybridization and immunostaining showed LINE1 was drastically up-regulated at both mRNA and protein levels in *Miwi*^*-/-*^ testes [27,41] (Figs 5A, 5B and S6). Surprisingly, despite the observed piRNA defects in *Miwi*^*RK/-*^ testes, *LINE1* mRNA and ORF1 protein expression remained suppressed in *Miwi*^*RK/-*^ testes, similar to *Miwi*^*+/-*^ controls (Figs 5A, 5B and S6). This suggests that the RK mutation does not interfere with MIWI slicer activity required for LINE1 silencing and infers that piRNAs against LINE1 are sufficiently produced in *Miwi*^*RK/-*^ germ cells. To confirm this, we analyzed LINE1-derived piRNAs in *Miwi*^*RK/-*^ testes. The Ping-Pong signature of *Miwi*^*RK/-*^ MIWI-piRNAs mapped to LINE1 was evident and comparable to that of *Miwi*^*+/-*^ (Fig 5C). The same Ping-Pong signature was observed in total piRNA and MILI-piRNAs in both *Miwi*^*RK/-*^ and *Miwi*^*+/-*^, indicating that Ping-Pong triggered LINE1 silencing is functional in *Miwi*^*RK/-*^ germ cells (S7A and S7B Fig). Consistent with this, the distribution of mapped sense and antisense LINE1 piRNAs from MIWI-piRNAs, MILI-piRNAs and total piRNAs was comparable between *Miwi*^*RK/-*^ and *Miwi*^*+/-*^ testes (Figs 5D, S7C and S7D). Additionally, we performed RT-qPCR to investigate whether *LINE1* and endogenous retroviruses (*IAP*, *MusD* and *MERVL*) were affected at the mRNA level in *Miwi*^*RK/-*^ testes. Consistent with *in situ* hybridization and immunostaining results, *LINE1* was up-regulated in *Miwi*^*-/-*^ testes but unchanged in *Miwi*^*RK/-*^ testes (S8 Fig). Endogenous retroviruses (*IAP*, *MusD* and *MERVL*) were not affected in both *Miwi*^*RK/-*^ and *Miwi*^*-/-*^ testes (S8 Fig). Collectively, we conclude that the RK mutation does not cause LINE1 de-repression, suggesting that germ cell arrest in *Miwi*^*RK/-*^ mice is not triggered by improper LINE1 activation.

### Chromatoid body malformation and round spermatid arrest in *Miwi*^*RK/-*^ mice are independent of DNA damage and apoptosis

**Fig 6:**
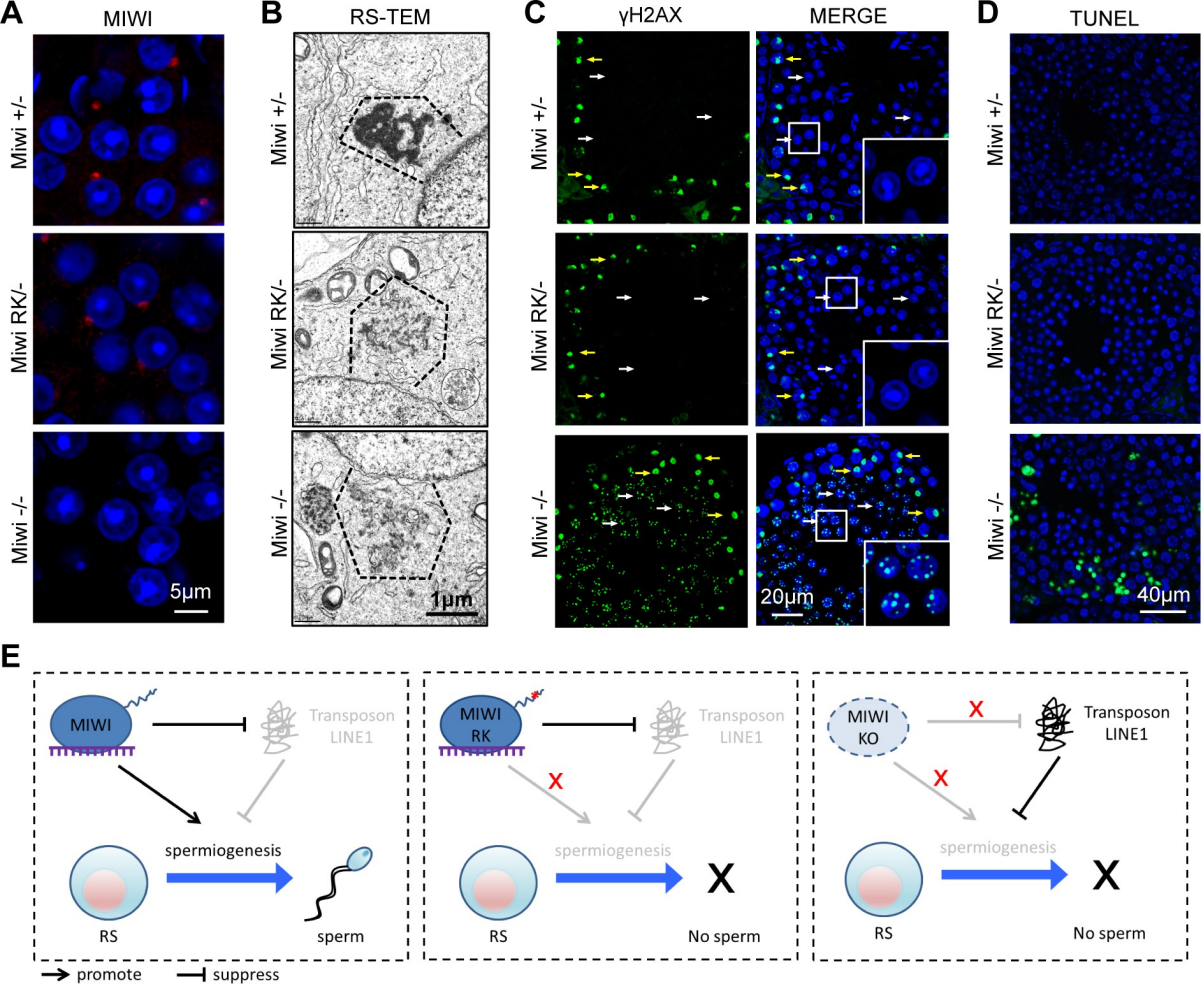
MIWI RK mutation causes chromatoid body malformation but does not induce DNA damage and apoptosis. **(A)** MIWI^RK^ localization to chromatoid body in round spermatids. Testes from indicated mice were immunostained using MIWI antibody. DNA was stained by DAPI. Scale bar, 5 μm. **(B)** The RK mutation in MIWI causes chromatoid body fragmentation in round spermatids. Transmission electron microscopy was performed on round spermatids (RS) from *Miwi*^*+/-*^, *Miwi*^*RK/-*^, and *Miwi*^*-/-*^ testes. The chromatoid bodies are indicated by dotted line. Scale bar, 1 μm. **(C)** MIWI RK mutation does not cause DNA damage in arrested round spermatids. Adult testes were immunostained using γH2AX antibody. DNA was stained by DAPI. Scale bar, 20 μm. **(D)** MIWI RK mutation does not cause apoptosis in testes. TUNEL assays were performed in adult testes. DNA was stained by DAPI. Scale bar, 40 μm. **(E)** A proposed model illustrating the role of the MIWI N-terminal RG motif in spermiogenesis. Results shown in (A)-(D) are representative of 3 biological replicates.

MIWI is predominately localized to the chromatoid body (CB) in round spermatids. We next examined the fate of MIWI^RK^ in *Miwi*^*RK/-*^ round spermatids. Despite the presence of MIWI^RK^ in the CB, transmission electron microscopy revealed CB fragmentation in *Miwi*^*RK/-*^ round spermatids (Fig 6A and 6B). The observed CB fragmentation is similar to *Miwi*^*-/-*^ and represents a common defect associated with low MIWI expression (Fig 6B). Thus, the RK mutation causes defective CB formation in round spermatids which can be explained by inefficient piRNA biogenesis.

*Miwi*^*-/-*^ round spermatids arrest at step 1–3 and undergo DNA damage and apoptosis [11]. We then investigated whether DNA damage and/or apoptosis contribute to the late step 8 round spermatid arrest observed in *Miwi*^*RK/-*^ testes. While γH2AX positive foci were abundant in *Miwi*^*-/-*^ round spermatids indicative of DNA damage, they were not present in *Miwi*^*RK/-*^ round spermatids (Fig 6C). TUNEL assay further showed no evident apoptosis in *Miwi*^*RK/-*^ round spermatids in contrast to elevated apoptosis observed in *Miwi*^*-/-*^ testes (Fig 6D). These data collectively indicate that despite causing piRNA defects, the RK mutation does not induce DNA damage or apoptosis in male germ cells, which is a significant difference from *Miwi*^*-/-*^ mice.

## Discussion

Our results provide the first evidence that an RG motif of a mammalian PIWI protein is functionally important for piRNA biogenesis, spermatogenesis, and male fertility. Importantly, the *Miwi*^*RK*^ mutant mouse model genetically decouples MIWI’s function in LINE1 transposon silencing and its role in promoting spermiogenesis (Fig 6E). MIWI N-terminal RG motif is required for efficient piRNA production, suggesting a critical role for this conserved motif in other vertebrate PIWIL1-like proteins to regulate the process of spermiogenesis, the last step of spermatogenesis in diverse species (S1A Fig).

MIWI N-terminal RG motif is required for MIWI-TDRKH interaction. We show here that MIWI-TDRKH interaction is disrupted by the *Miwi*^*RK*^ mutation in germ cells. Consequently, MIWI^RK^ fails to be efficiently recruited to the IMC and MIWI^RK^-bound piRNA production is much reduced. Despite a reduction in piRNA levels, piRNA processing and trimming are complete because there were no obvious length changes for MIWI^RK^-bound piRNAs, suggesting that MIWI^RK^ can engage in piRNA processing and trimming from start to finish. This is consistent with our observation that the RK mutation in MIWI does not affect TDRKH anchoring on the mitochondrial surface for piRNA trimming. The accessibility of MIWI^RK^ to the piRNA trimming complex can be explained by its residual weak interaction with TDRKH or its direct association with the trimmer PNLDC1 [43,44]. Alternatively, other piRNA biogenesis factors may compensate for the recruitment of MIWI^RK^ to the IMC for piRNA processing. Nonetheless, mature piRNAs associated with MIWI^RK^ still follow the abundance pattern derived from piRNA clusters found in wild-type germ cells, indicating it is the piRNA production efficiency but not piRNA precursor selectivity that is affected by the RK mutation.

One surprising finding is that the MIWI N-terminal RG motif is required for primary piRNA biogenesis but not required for LINE1 silencing. This genetically separates MIWI’s transposon silencing function from its ability to promote spermiogenesis. The maintenance of LINE1 silencing in *Miwi*^*RK*^ mutants is likely due to the slightly reduced but functional LINE1-targeting piRNAs and the normal Ping-Pong signature in mutant germ cells, which differs from *Miwi*^*-/-*^ and MIWI slicer mutants that both show significant LINE1 activation [27]. Thus, the round spermatid arrest in *Miwi*^*RK/-*^ mutants can be dissociated from LINE1 silencing and possibly results from reduction of the bulk of pachytene piRNAs primarily derived from transposon-poor pachytene piRNA clusters. The fragmented CB in *Miwi*^*RK/-*^ round spermatids can also be ruled out to be a direct consequence of defective LINE1 silencing. In another genetic model, conditional deletion of piRNA biogenesis factor *Mov10l1* by Stra8-Cre causes severe reduction in both MILI-piRNAs and MIWI-piRNAs and spermiogenic arrest without impacting LINE1 silencing, suggesting the critical roles of non-transposon-targeting pachytene piRNAs in promoting spermiogenesis [37].

MIWI/PIWIL1’s overall function is carried out by the coordinated actions of its functional domains. The RK mutation abolishes the function of the N-terminal RG motif, while the folding and function of other major domains, PAZ, MID, and PIWI, are not expected to be altered. Indeed, the piRNA-binding ability mediated by the PAZ and MID domains of MIWI^RK^ is unaffected by the RK mutation. The slicer activity conferred by the PIWI domain is also retained by MIWI^RK^ as LINE1 remains silenced in *Miwi*^*RK/-*^ mice. A D-box motif in the N-terminal region of MIWI involving ubiquitination has been shown to be important for male fertility [45,46]. Mice with D-box mutations display a late spermiogenesis defect differing from that of *Miwi*^*RK/-*^ mice, highlighting the designated roles of various functional domains/motifs in directing MIWI function during spermatogenesis.

PIWI proteins interact with the TDRD family proteins through arginine-rich motifs and the PIWI-TDRD interaction is conserved among species. However, the binding mechanism varies across species. In mice, TDRKH interacts with the MIWI N-terminal RG motif independent of arginine methylation [28,40]. While in *Drosophila*, Papi, the homolog of TDRKH, interacts with Piwi in an arginine methylation-dependent manner [47]. *Drosophila* PIWI protein Aub interacts with Tudor domain-containing proteins Tud and Krimper through a N-terminal RG motif similar to that found in mouse MIWI2 (PIWIL4) [48,49]. By replacing the four arginines in the N-terminal RG motif to lysines, the Aub RK mutant flies show high embryonic lethality and transposon upregulation but intact primary piRNA biogenesis [50,51]. By contrast, here we demonstrate that the mouse MIWI N-terminal RG motif is required for spermiogenesis by promoting piRNA biogenesis independent of LINE1 silencing. Our results highlight the functional heterogeneity of RG motifs in different PIWI proteins across diverse species. In mice, the function of the RG motifs in MILI and MIWI2 to regulate piRNA biogenesis, spermatogenesis, and fertility requires further investigation.

## Materials and methods

### Ethics statement

All animal procedures were approved by the Institutional Animal Care and Use Committee of Michigan State University. All experiments with mice were conducted ethically according to the Guide for the Care and Use of Laboratory Animals and institutional guidelines.

### Generation of *Miwi*^*RK*^ mutant mice

*Miwi*^*RK*^ mutant mice were generated by CRISPR-Cas9 targeting of the mouse *Piwil1* locus (ENSMUSG00000029423). Wild-type NLS-Cas9 protein, synthetic tracrRNA, crRNA and a single-stranded oligodeoxynucleotide (ssODN) donor template were purchased from Integrated DNA Technologies (Coralville, IA, USA). Protospacer (N)_20_ and *PAM* sequences corresponding to the crRNA used were 5’-TGACTGGCCGAGCCCGAGCT *-CGG* -3’. Donor ssODNs in the reverse orientation had the following sequence: 5’ AGCTATATAAGAATGGTACTCACCGCAGCAGCCCCAACATGCTGCACCGTCTCCTGACCcttTGCCtTGCCcttGGCCttAGCcttGGCcttGCCAGTCATTTTCTGTCAGAGAGGAAAAGCACACGA 3’. Synthetic tracrRNA and crRNA were incubated at 95°C for 5 min and allowed to cool down in order to form RNA heteroduplexes, which were then incubated with Cas9 protein for 5 min at 37°C to pre-form ribonucleoprotein (RNP) complexes. RNPs were electroporated into mouse zygotes using a Gene Editor electroporator (BEX CO., LTD, Tokyo, Japan) as previously described [52]. Embryos were implanted into pseudo-pregnant recipients according to standard procedures. Editing of founder offspring was assessed using PCR, T7 Endonuclease I assay, and Sanger sequencing of the target region.

Miwi knockout mice (*Miwi*^*-/-*^), generated in the laboratory of Dr. Haifan Lin [11], were purchased from Mutant Mouse Resource Research Centers.

### Plasmid construction

The full-length mouse *Tdrd1*, *Rnf17*, *Tdrd5*, *Tdrd6*, *Stk31*, *Pnldc1* and *Mov10l1* cDNAs were amplified by PCR and cloned into pEGFP-C1 (GFP tag at N-terminus) expression vector. The full-length mouse *Tdrkh* cDNA was amplified by PCR and cloned into pEGFP-N1 (GFP tag at C-terminus) expression vector. The full-length mouse *Miwi* and *Miwi*^*RK*^ mutant cDNAs were amplified by PCR and cloned into pcDNA3-FLAG (FLAG tag at N-terminus) and pEGFP-C1 (GFP tag at N-terminus) expression vectors. To obtain RFP-tagged TDRKH, the GFP tag of pEGFP-N1 vector was replaced by TurboRFP tag and the full-length mouse *Tdrkh* cDNA was cloned into this TurboRFP-tagged (TurboRFP tag at C-terminus) expression vector.

### Histology

Mouse testes and epididymides were collected and fixed in Bouin’s fixative at 4°C overnight and embedded in paraffin. Histological sections were cut at 5 μm and stained with hematoxylin and eosin after dewaxing and rehydration.

### Immunofluorescence

Mouse testes were fixed in 4% paraformaldehyde (PFA) overnight at 4°C and embedded in paraffin. Tissue sections were cut at 5 μm, dewaxed and rehydrated. Antigen retrieval was performed in Tris-EDTA buffer (pH 9.0). Testis sections were blocked in 5% NGS for 30 min at room temperature. Testis sections were then incubated with anti-MIWI (1:100; 2079, Cell Signaling Technology), anti-MILI (1:100; PM044, MBL), anti-TDRKH (1:100; 13528-1-AP, Proteintech), anti-α-tubulin (1:100; sc32293, Santa Cruz), anti-ACRV1 (1:50; 14040-1-AP, Proteintech), anti-LINE1 ORF1 (1:800), or FITC-conjugated mouse anti-γH2AX (1:500; 16-202A, Millipore) in 5% NGS at 37°C for 2 h. After washing with PBS, sections were incubated with Alexa Fluor 555 goat anti-rabbit IgG (1:500; A21429, Life Technologies) for 1 h and mounted using Vectashield mounting media with DAPI. For MIWI and TDRKH colocalization, MIWI staining was performed as above, followed by incubating with fluorescently labeled anti-TDRKH (1:20) for 2 h at room temperature. Anti-TDRKH was labeled by Zenon Alexa Fluor 488 Rabbit IgG Labeling Kit (Z25302, Thermo Scientific) according to manufacturer’s instruction. For MIWI and TOMM20 colocalization, sections were incubated with anti-TOMM20 (1:100; ab186734, Abcam) and then incubated with Alexa Fluor 488 goat anti-rabbit IgG (1:500; A11034, Life Technologies), followed by incubating with fluorescently labeled anti-MIWI (1:20) for 2 h at room temperature. Anti-MIWI was labeled by Zenon Alexa Fluor 555 Rabbit IgG Labeling Kit (Z25305, Thermo Scientific) according to manufacturer’s instruction. Fluorescence microscopy was performed using Fluoview FV1000 confocal microscope.

### TUNEL assay

To evaluate apoptosis, TUNEL assay was performed on testis sections using *In Situ* Cell Death Detection Kit (11684795910, Sigma-aldrich) according to manufacturer’s instruction. Briefly, 5 μm PFA-fixed paraffin sections were dewaxed and rehydrated, followed by incubating with Proteinase K for 20 min at room temperature. After washing with PBS, sections were incubated with TUNEL reaction mixture at 37°C for 1 h and mounted using Vectashield mounting media with DAPI.

### Western blotting

Mouse testes were collected and homogenized in RIPA buffer (50 mM Tris-HCl pH 7.4, 1% NP-40, 0.5% sodium deoxycholate, 0.01% SDS, 1 mM EDTA, and 150 mM NaCl) with protease inhibitor. Protein lysates were separated by 4–20% SDS-PAGE gel and transferred to PVDF membranes. The membranes were blocked in 5% non-fat milk and subsequently incubated with primary antibodies in blocking solution overnight at 4°C. Membranes were washed with TBST and incubated with HRP-conjugated goat anti-rabbit IgG (1:5000; 1706515, Bio-Rad) or goat anti-mouse IgG (1:5000; 1706516, Bio-Rad) for 1 h at room temperature before chemiluminescent detection. The primary antibodies used were anti-TDRKH (1:4000; 13528-1-AP, Proteintech), anti-MIWI (1:1000; 2079, Cell Signaling Technology), anti-MILI (1:2000; PM044, MBL), anti-TDRD6 (1:1000) and HRP-conjugated mouse anti-β-actin (1:5000; A3854, Sigma).

### Cell transfection and co-immunoprecipitation

HEK293T cells were transfected with indicated plasmids using Lipofectamine 2000 (Life Technologies). After 48 h, immunoprecipitation was performed using anti-FLAG M2 Affinity Gel (A2220, Sigma). FLAG-tagged or GFP-tagged proteins were detected by Western blotting using anti-FLAG antibody (1:1000; F1804, Sigma) or anti-GFP antibody (1:10000; Ab290, Abcam) and secondary antibodies HRP-conjugated goat anti-mouse IgG (1:5000; 1706516, Bio-Rad) or goat anti-rabbit IgG (1:5000; 1706515, Bio-Rad). HeLa cells were transfected with indicated plasmids using Lipofectamine 2000 (Life Technologies). After 48 h, cells were fixed with 4% PFA and DNA was stained with DAPI. Fluorescence microscopy was performed using Fluoview FV1000 confocal microscope.

### Transmission electron microscopy

Mouse testes were fixed with 2.5% glutaraldehyde and 2% PFA in 0.1 M cacodylate buffer for 2 h at room temperature. After washing, the testes were post-fixed with 1% osmium tetroxide in 0.1 M cacodylate buffer for 2 h at room temperature. The testes were dehydrated in increasing concentrations of acetone and then infiltrated and embedded in Spurr’s resin. Ultrathin sections were cut at 70 nm and stained with uranyl acetate and lead citrate. Images were taken with JEOL 100CX Transmission Electron Microscope (Japan Electron Optics Laboratory, Japan) at an accelerating voltage of 100kV.

### Immunoprecipitation of piRNAs and proteins

Mouse testes were collected and homogenized using lysis buffer (20 mM HEPES pH 7.3, 150 mM NaCl, 2.5 mM MgCl_2_, 0.2% NP-40, and 1 mM DTT) with protease inhibitor and RNase inhibitor. The lysates were pre-cleared using protein-A agarose beads for 2 h at 4°C. Anti-MILI (PM044, MBL) or anti-MIWI (2079, Cell Signaling Technology) antibodies together with protein-A agarose beads were added to the lysates and incubated for 4 h at 4°C. The beads were washed in lysis buffer 5 times. Immunoprecipitated RNAs were isolated from the beads using Trizol reagent for piRNA labeling or small RNA library construction. For protein detection, immunoprecipitated beads were boiled in protein loading buffer for 5 min. Western blotting of MILI, MIWI, TDRKH or TDRD6 was performed as described above.

### Detection of piRNAs

Total RNA was extracted from mouse testes using Trizol reagent (Thermo Scientific). Total RNA or immunoprecipitated RNA (MILI or MIWI) was de-phosphorylated with Shrimp Alkaline Phosphatase (NEB) and end-labeled using T4 polynucleotide kinase (NEB) and [γ-32P] ATP. The 32P-labeled RNA was separated on a 15% Urea-PAGE gel, and signals were detected by exposing the gel on phosphorimager screen followed by scanning on the Typhoon scanner (GE Healthcare).

### Small RNA libraries and bioinformatics

Small RNA libraries from immunoprecipitated RNAs or total RNA were prepared using Small RNA Library Prep Kit (E7300, NEB) following manufacturer’s instructions. Multiple libraries with different barcodes were pooled and sequenced with the Illumina HiSeq 4000 platform (MSU Genomic Core Facility).

Sequenced reads were processed with fastx_clipper (http://hannonlab.cshl.edu/fastx_toolkit/index.html) to clip the sequencing adapter read-through. Clipped reads were filtered by length (24–32 nt) and aligned to the following sets of sequences: piRNA clusters, coding RNAs, non-coding RNAs, repeats, introns, and other. Alignments were performed with Bowtie (one base mismatch allowed). Repeats included classes of repeats as defined by RepeatMasker (ftp://hgdownload.cse.ucsc.edu/goldenPath/mm10/database/rmsk.txt.gz).

### *In situ* hybridization

Testes were fixed in 4% PFA overnight at 4°C. After being immersed in 30% sucrose, testes were embedded in O.C.T compound and frozen before 7 μm sections were cut. Antisense DIG labeled RNA probe was transcribed using DIG RNA Labeling Mix (Roche) from a linearized plasmid containing a full length of *LINE1 Orf1* (nucleotides 1741–2814, GenBank: M13002.1). After denaturing the probes for 10 min in hybridization cocktail solution (Amresco), the probes were added to the sections and incubated overnight at 65°C. After washing and blocking, sections were incubated with alkaline-phosphatase conjugated goat anti-DIG Fab fragments (Roche) overnight. The positive signal was visualized by adding BM Purple (Roche).

### RT-qPCR

Total RNA was extracted from mouse testes using Trizol reagent (Thermo Scientific) and treated with TURBO DNA-free Kit (AM1907, Thermo Scientific). 1 μg treated RNA was reverse transcribed using iScript cDNA Synthesis Kit (1708890, Bio-Rad) according to manufacturer’s instructions. cDNA was diluted 4-fold and qPCR was performed using iTaq Universal SYBR Green Supermix (1725121, Bio-Rad) in QuantStudio 5 Real-Time PCR System (Thermo Scientific). qPCR conditions were 95°C for 10 min, followed by 40 cycles of 95°C for 15 s and 60°C for 60 s. Primers used for qPCR are: *LINE1* forward 5’- GGAGGGACATTTCATTCTCATCA-3’, *LINE1* reverse 5’- GCTGCTCTTGTATTTGGAGCATAGA-3’; *IAP* forward 5’- AACCAATGCTAATTTCACCTTGGT-3’, *IAP* reverse 5’- GCCAATCAGCAGGCGTTAGT-3’; *MusD* forward 5’- GTGGTATCTCAGGAGGAGTGCC-3’, *MusD* reverse 5’- GGGCAGCTCCTCTATCTGAGTG-3’; *MERVL* forward 5’- CTTCCATTCACAGCTGCGACTG-3’, *MERVL* reverse 5’- CTAGAACCACTCCTGGTACCAAC-3’; *Gapdh* forward 5’- AGAAACCTGCCAAGTATGATGAC-3’, *Gapdh* reverse 5’- GTCATTGAGAGCAATGCCAG-3’.

### Statistical analysis

All data are mean±SEM and all statistical analyses between groups were analyzed by unpaired t-test.

## Supporting information

S1 Fig The generation of *Miwi*^*RK*^ mutant mice. **(A)** Multiple sequence alignment of PIWI N-terminal RG motifs from PIWI proteins of different species. **(B)** A schematic diagram showing the strategy for the generation of a *Miwi*^*RK*^ allele using CRISPR-Cas9. **(C)** The RK mutation was shown by DNA sequencing.
(TIF)

S2 Fig Phagocytic removal of multinucleated giant cells in *Miwi*^*RK/-*^ testes by Sertoli cells. Testes were immunostained using α-tubulin antibody. The multinucleated giant cells in *Miwi*^*RK/-*^ testes are indicated by white arrows. Scale bar, 40 μm. Results shown are representative of 3 biological replicates.
(TIF)

S3 Fig MIWI RK mutation causes mislocalization of MIWI during spermatogenesis in mice. **(A)** Testes from indicated mice were immunostained using MIWI and γH2AX antibodies. DNA was stained by DAPI. Different spermatogenic stages were distinguished according to γH2AX staining and DAPI staining. Scale bar, 40 μm. **(B)** The RK mutation diminishes the recruitment of MIWI to mitochondria in pachytene spermatocytes. Co-immunostaining of MIWI and mitochondrial marker TOMM20 in stage VII–VIII seminiferous tubule from *Miwi*^*+/-*^ and *Miwi*^*RK/*-^ testes was performed. DNA was stained by DAPI. Scale bar, 10 μm. Results shown in (A) and (B) are representative of 3 biological replicates.
(TIF)

S4 Fig MIWI RK mutation does not affect the expression and localization of MILI and TDRKH. **(A)** Expressions of MIWI, MILI, and TDRKH in adult testes are revealed by Western blotting. β-actin is a loading control. Quantification of intensity of MIWI is shown under the blot (the one in wildtype testis is set as 1.000 after normalization with β-actin). **(B)** Testes from indicated mice were immunostained using MILI or TDRKH antibodies. DNA was stained by DAPI. Scale bar, 20 μm. Results shown in (A) and (B) are representative of 3 biological replicates.
(TIF)

S5 Fig MIWI N-terminal RG motif is required for MIWI-piRNA biogenesis from piRNA clusters. **(A)** The number of piRNA reads mapped to Top50 piRNA clusters from total piRNAs were shown. piRNA reads were normalized by the miRNA counts of each small RNA library. n = 2. Error bars represent s.e.m. **(B)** The number of piRNA reads mapped to Top50 piRNA clusters from MILI-piRNAs were shown. The data were normalized by total small RNA reads from each library. n = 2. Error bars represent s.e.m. **(C)** The number of piRNA reads mapped to Top50 piRNA clusters from MIWI-piRNAs were shown. The data were normalized by total small RNA reads from each library. n = 2. Error bars represent s.e.m.
(TIF)

S6 Fig MIWI RK mutation does not cause LINE1 de-repression. Immunostaining was performed using LINE1 ORF1 antibody on adult testes. DNA was stained with DAPI. Scale bar, 100 μm. Results shown are representative of 3 biological replicates.
(TIF)

S7 Fig MIWI RK mutation does not affect the Ping-Pong signature of LINE1-derived piRNAs. **(A)** The 5′-5′ overlaps between total piRNAs from opposite strands of LINE1 elements from *Miwi*^*+/-*^ and *Miwi*^*RK/-*^ testes are shown. The percentage of pairs of piRNA reads at each position is reported. **(B)** The 5′-5′ overlaps between MILI-piRNAs from opposite strands of LINE1 elements from *Miwi*^*+/-*^ and *Miwi*^*RK/-*^ testes are shown. The percentage of pairs of piRNA reads at each position is reported. **(C)** Graphs show the distribution of total piRNAs that were mapped in the sense and antisense orientation to LINE1. piRNA reads were normalized by the miRNA counts of each small RNA library. **(D)** Graphs show the distribution of MILI-piRNAs that were mapped in the sense and antisense orientations to LINE1. Reads were normalized by the total reads from each library. Small RNA-seq results shown in (C) and (D) are representative of 2 biological replicates.
(TIF)

S8 Fig MIWI RK mutation does not cause *LINE1* de-repression. RT-qPCR analysis of *Miwi*^*+/-*^, *Miwi*^*RK/-*^ and *Miwi*^*-/-*^ testes for the expression of retrotransposons (*LINE1*, *IAP*, *MusD* and *MERVL*). n = 3. Error bars represent s.e.m. The P-value was calculated using unpaired t-test. *, P < 0.01.
(TIF)
