# Supplementary figures and images for: MIWI N-terminal RG motif promotes efficient pachytene piRNA production and spermatogenesis independent of LINE1 transposon silencing
Source: PLoS Genet. 2023 Nov 13;19(11):e1011031. doi: 10.1371/journal.pgen.1011031 (PMC10681313; doi:10.1371/journal.pgen.1011031)

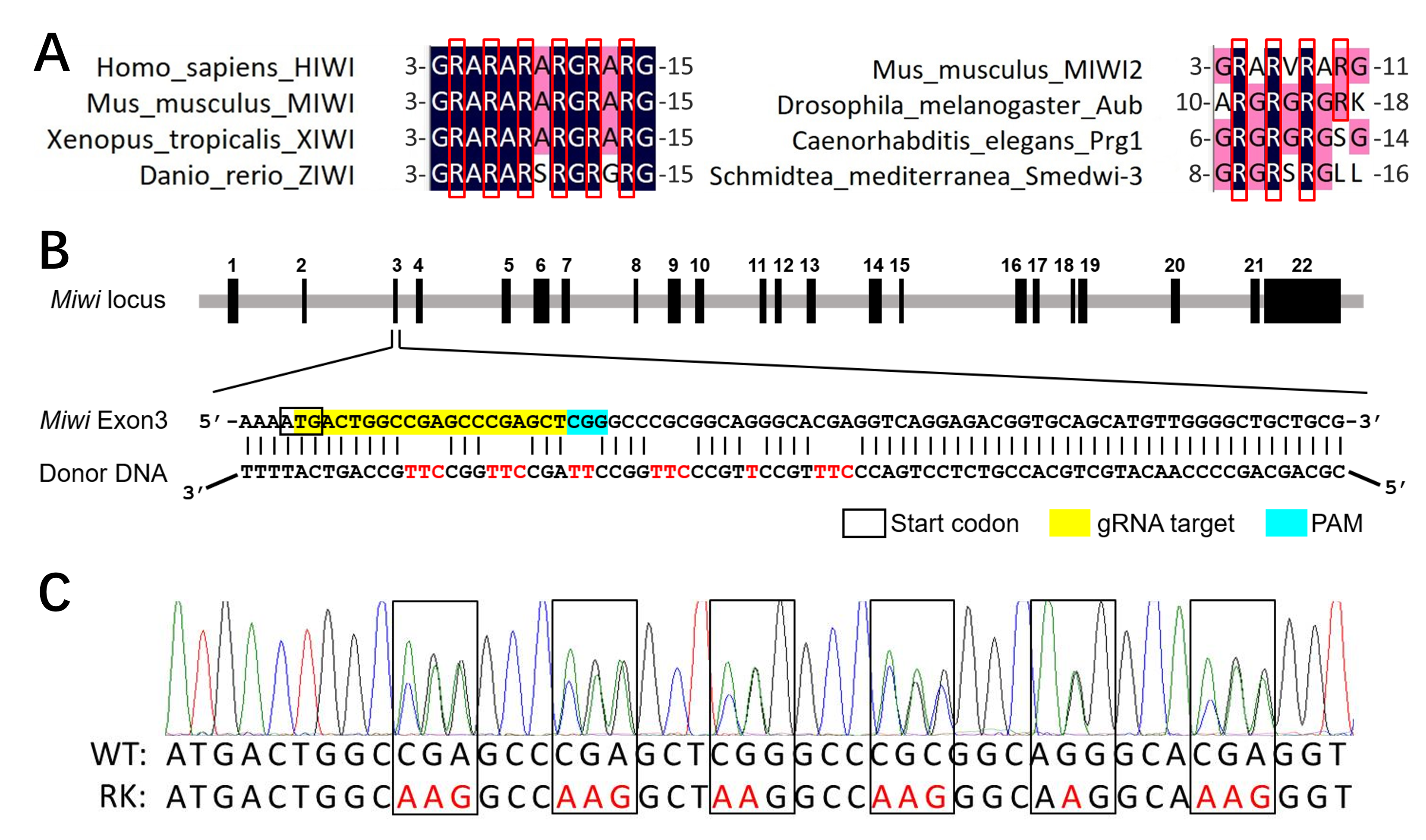

Supplement: S1 Fig — (A) Multiple sequence alignment of PIWI N-terminal RG motifs from PIWI proteins of different species. (B) A schematic diagram showing the strategy for the generation of a MiwiRK allele using CRISPR-Cas9. (C) The RK mutation was shown by DNA sequencing. (TIF) [file pgen.1011031.s001.tif]

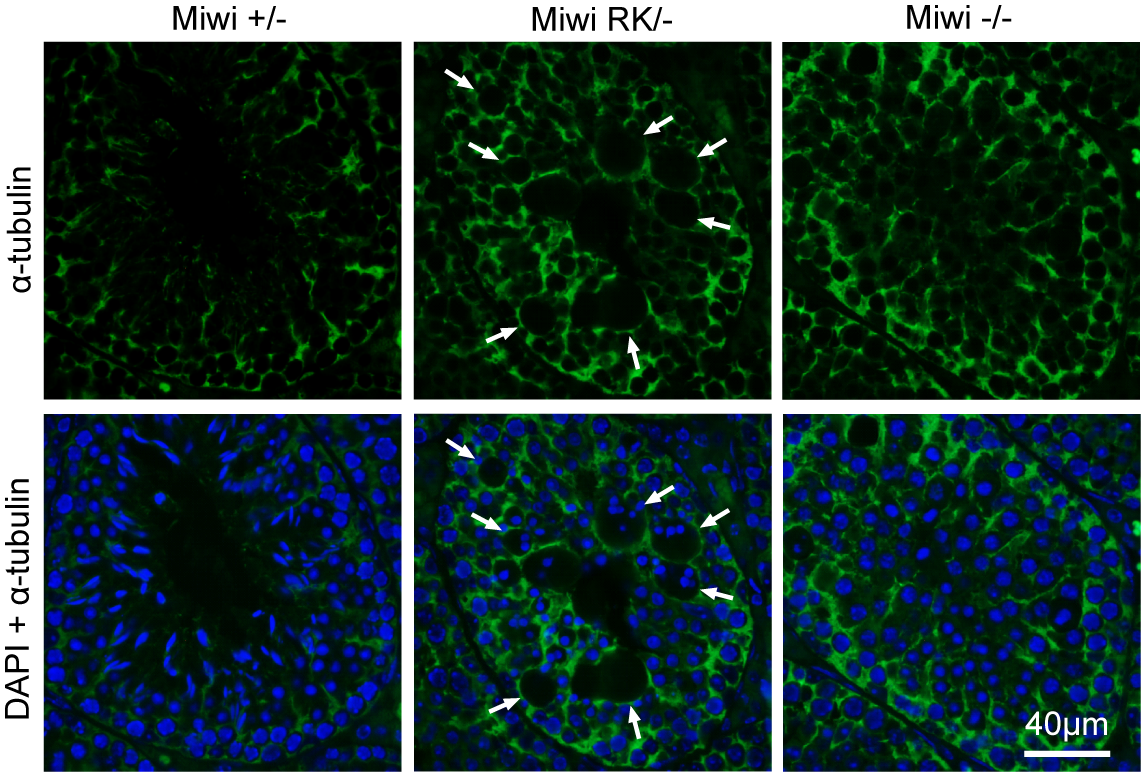

Supplement: S2 Fig — Testes were immunostained using α-tubulin antibody. The multinucleated giant cells in MiwiRK/- testes are indicated by white arrows. Scale bar, 40 μm. Results shown are representative of 3 biological replicates. (TIF) [file pgen.1011031.s002.tif]

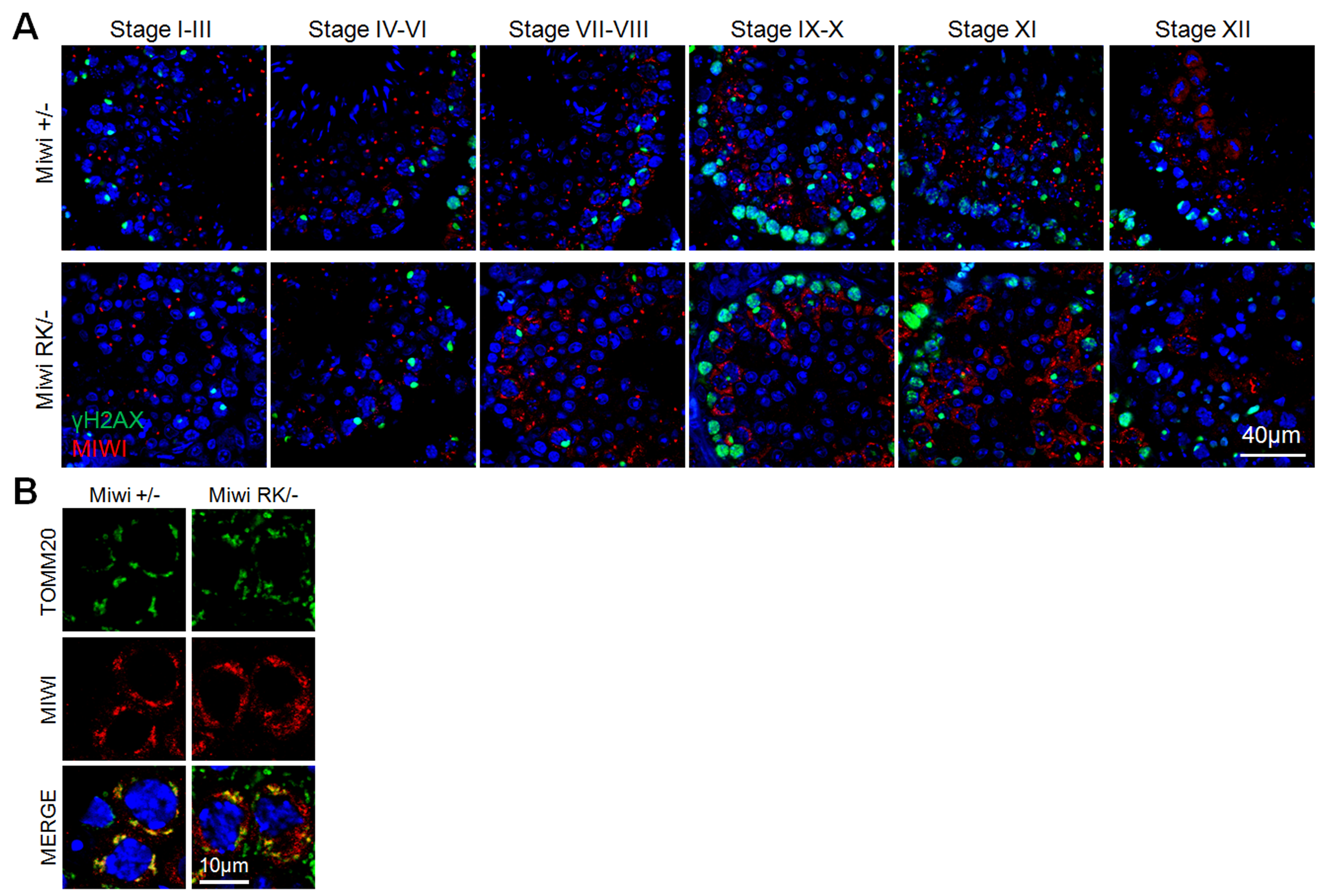

Supplement: S3 Fig — (A) Testes from indicated mice were immunostained using MIWI and γH2AX antibodies. DNA was stained by DAPI. Different spermatogenic stages were distinguished according to γH2AX staining and DAPI staining. Scale bar, 40 μm. (B) The RK mutation diminishes the recruitment of MIWI to mitochondria in pachytene spermatocytes. Co-immunostaining of MIWI and mitochondrial marker TOMM20 in stage VII–VIII seminiferous tubule from Miwi+/- and MiwiRK/- testes was performed. DNA was stained by DAPI. Scale bar, 10 μm. Results shown in (A) and (B) are representative of 3 biological replicates. (TIF) [file pgen.1011031.s003.tif]

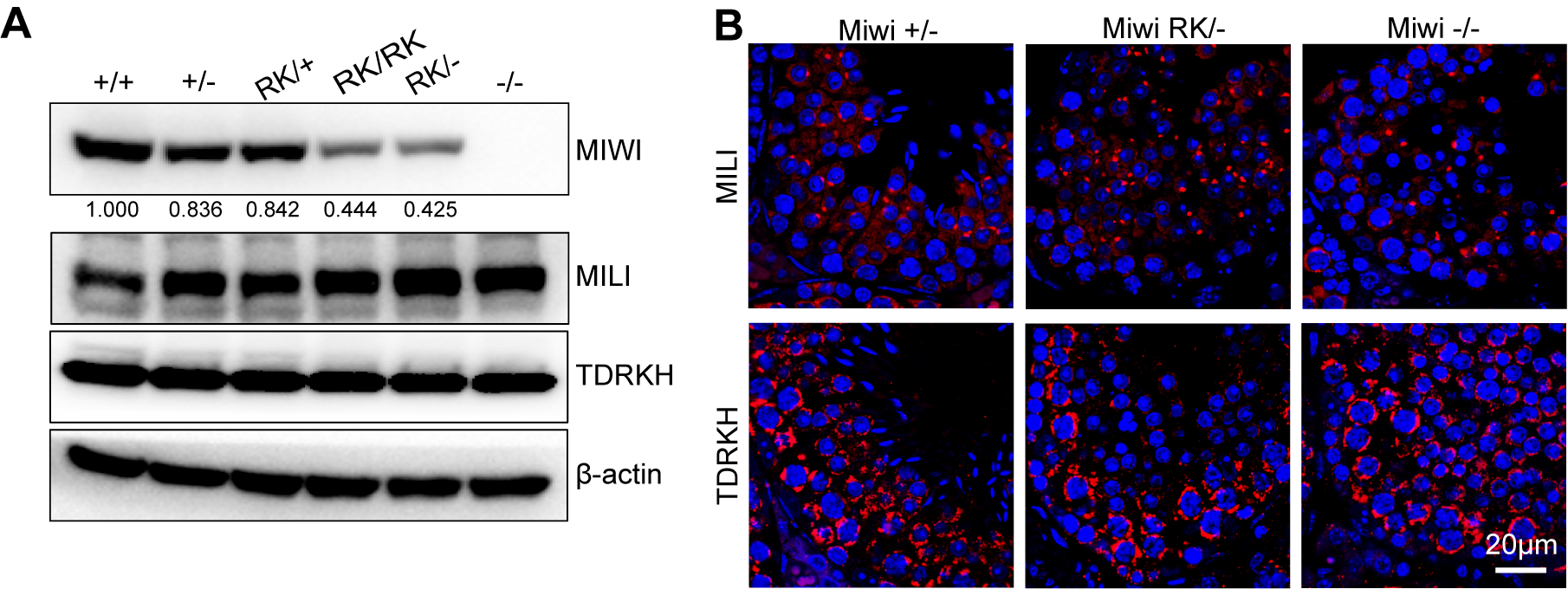

Supplement: S4 Fig — (A) Expressions of MIWI, MILI, and TDRKH in adult testes are revealed by Western blotting. β-actin is a loading control. Quantification of intensity of MIWI is shown under the blot (the one in wildtype testis is set as 1.000 after normalization with β-actin). (B) Testes from indicated mice were immunostained using MILI or TDRKH antibodies. DNA was stained by DAPI. Scale bar, 20 μm. Results shown in (A) and (B) are representative of 3 biological replicates. (TIF) [file pgen.1011031.s004.tif]

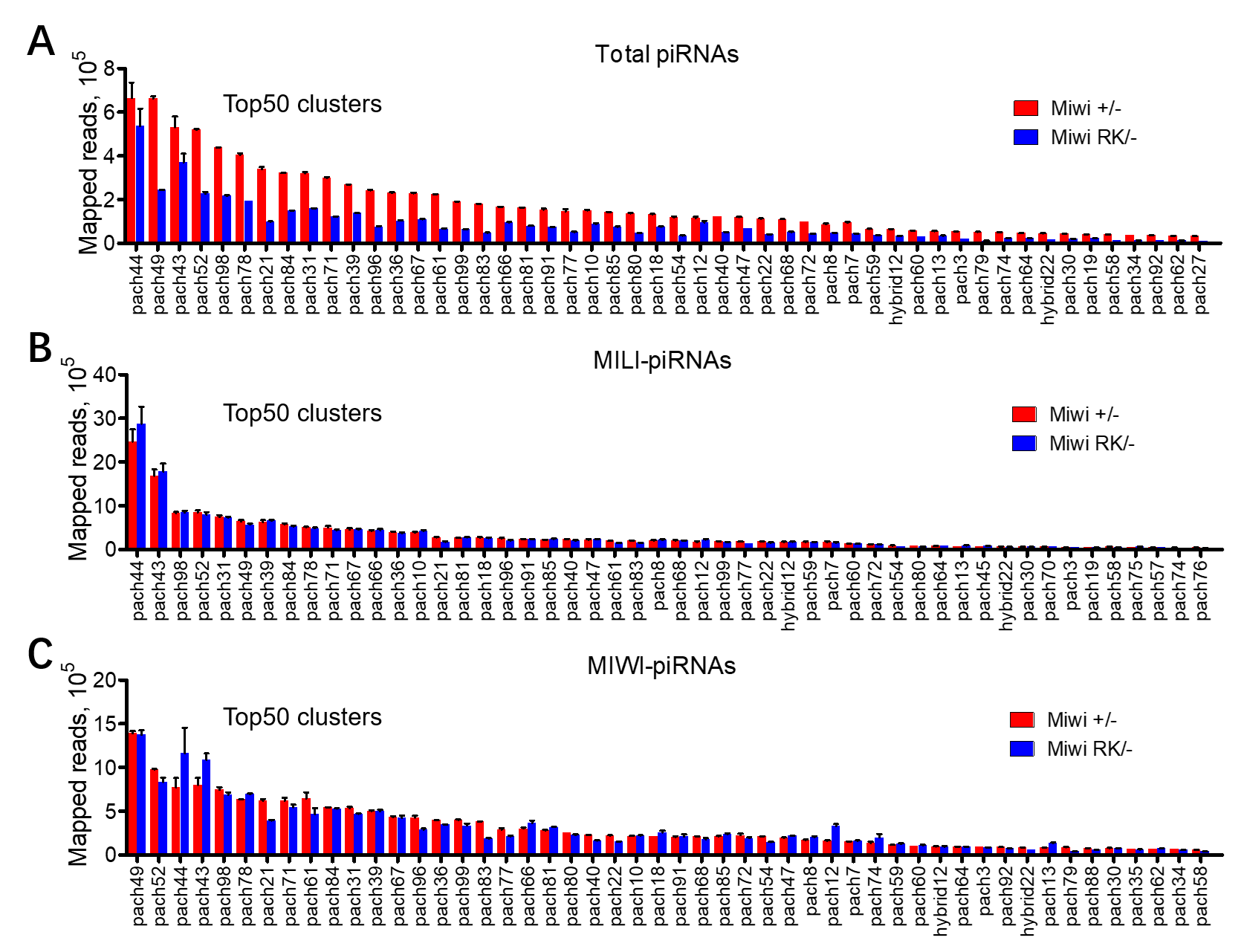

Supplement: S5 Fig — (A) The number of piRNA reads mapped to Top50 piRNA clusters from total piRNAs were shown. piRNA reads were normalized by the miRNA counts of each small RNA library. n = 2. Error bars represent s.e.m. (B) The number of piRNA reads mapped to Top50 piRNA clusters from MILI-piRNAs were shown. The data were normalized by total small RNA reads from each library. n = 2. Error bars represent s.e.m. (C) The number of piRNA reads mapped to Top50 piRNA clusters from MIWI-piRNAs were shown. The data were normalized by total small RNA reads from each library. n = 2. Error bars represent s.e.m. (TIF) [file pgen.1011031.s005.tif]

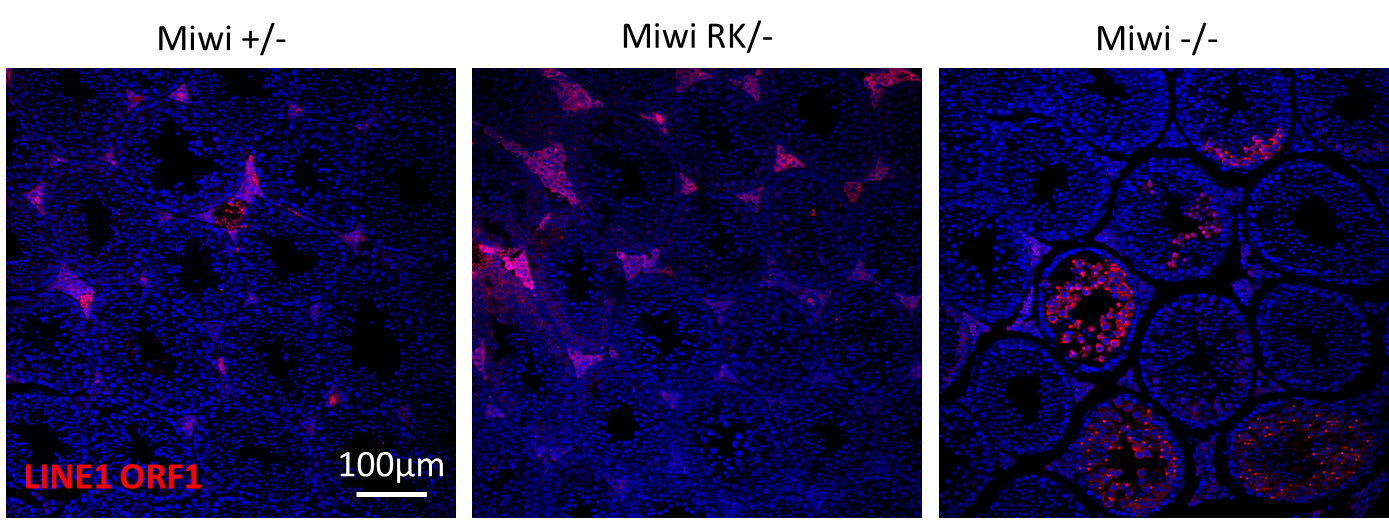

Supplement: S6 Fig — Immunostaining was performed using LINE1 ORF1 antibody on adult testes. DNA was stained with DAPI. Scale bar, 100 μm. Results shown are representative of 3 biological replicates. (TIF) [file pgen.1011031.s006.tif]

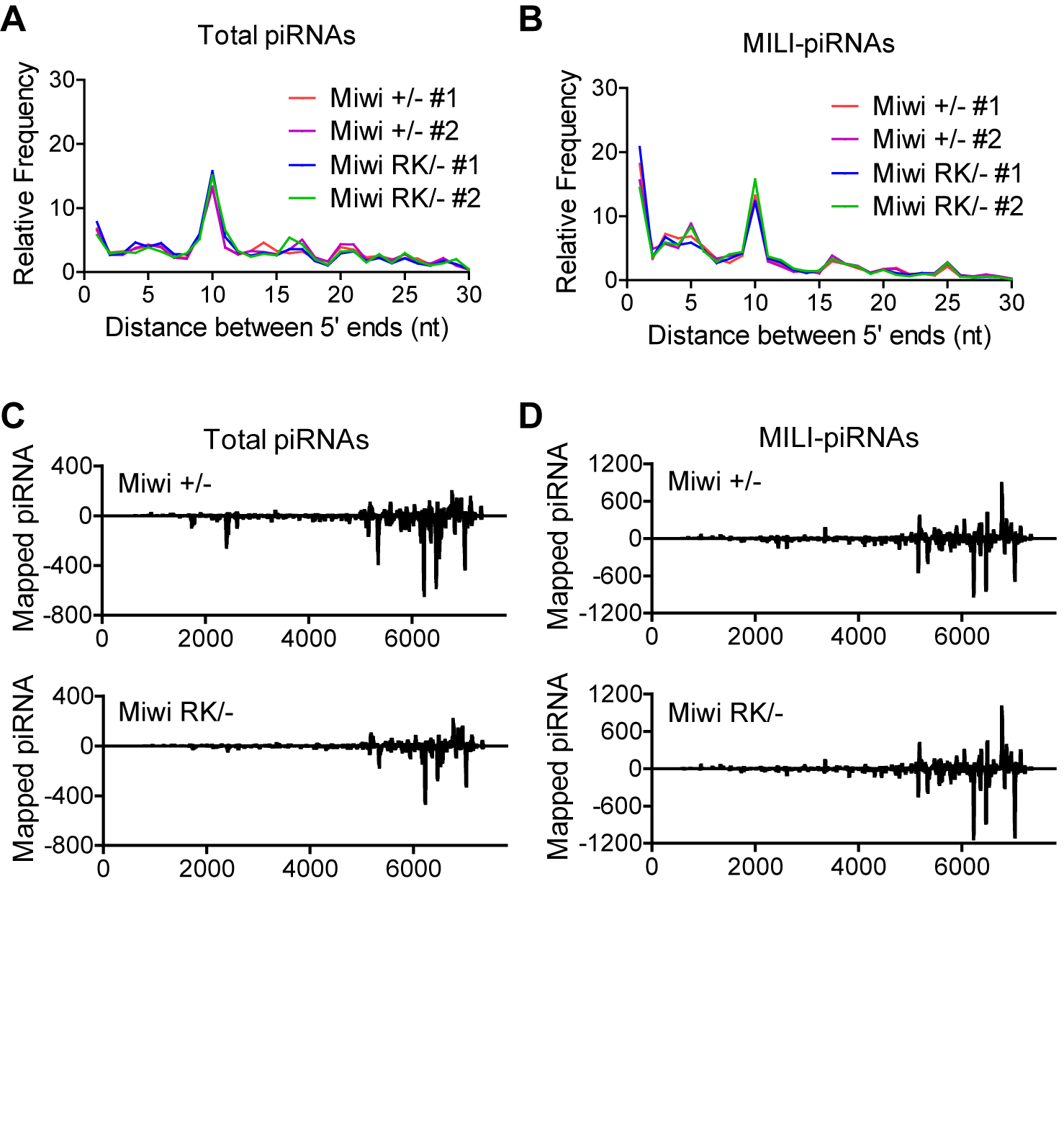

Supplement: S7 Fig — (A) The 5′-5′ overlaps between total piRNAs from opposite strands of LINE1 elements from Miwi+/- and MiwiRK/- testes are shown. The percentage of pairs of piRNA reads at each position is reported. (B) The 5′-5′ overlaps between MILI-piRNAs from opposite strands of LINE1 elements from Miwi+/- and MiwiRK/- testes are shown. The percentage of pairs of piRNA reads at each position is reported. (C) Graphs show the distribution of total piRNAs that were mapped in the sense and antisense orientation to LINE1. piRNA reads were normalized by the miRNA counts of each small RNA library. (D) Graphs show the distribution of MILI-piRNAs that were mapped in the sense and antisense orientations to LINE1. Reads were normalized by the total reads from each library. Small RNA-seq results shown in (C) and (D) are representative of 2 biological replicates. (TIF) [file pgen.1011031.s007.tif]

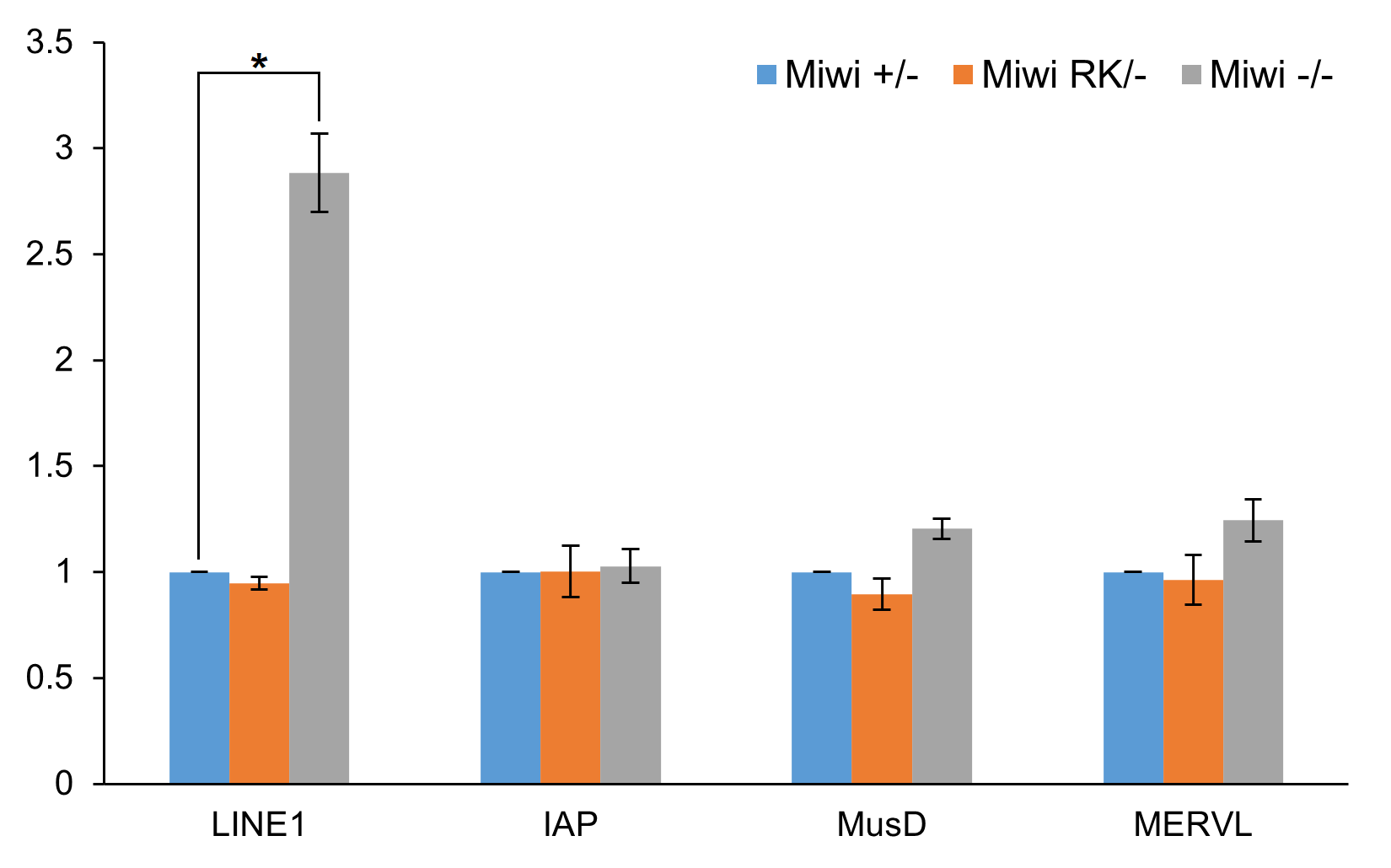

Supplement: S8 Fig — RT-qPCR analysis of Miwi+/-, MiwiRK/- and Miwi-/- testes for the expression of retrotransposons (LINE1, IAP, MusD and MERVL). n = 3. Error bars represent s.e.m. The P-value was calculated using unpaired t-test. *, P < 0.01. (TIF) [file pgen.1011031.s008.tif]
